# NfiR, a New Regulatory Noncoding RNA (ncRNA), Is Required in Concert with the NfiS ncRNA for Optimal Expression of Nitrogenase Genes in Pseudomonas stutzeri A1501

**DOI:** 10.1128/AEM.00762-19

**Published:** 2019-07-01

**Authors:** Yuhua Zhan, Zhiping Deng, Yongliang Yan, Hongyang Zhang, Chao Lu, Zhimin Yang, Liguo Shang, Yi Huang, Fanyang Lv, Yaqun Liu, Yichao Liu, Shanshan Wang, Sanfeng Chen, Xue-Xian Zhang, Qi Cheng, Min Lin

**Affiliations:** a Biotechnology Research Institute/National Key Facility for Crop Gene Resources and Genetic Improvement, Chinese Academy of Agricultural Sciences, Beijing, China; b Shenyang Medical College, Shenyang, China; c Key Laboratory for Agrobiotechnology, Ministry of Agriculture, China Agricultural University, Beijing, China; d Institute of Natural and Mathematical Sciences, Massey University at Albany, Auckland, New Zealand; University of California, Davis

**Keywords:** NfiR, *Pseudomonas stutzeri*, *nifD* mRNA, nitrogen fixation, regulatory ncRNA

## Abstract

Expression of nitrogenase genes (*nifHDK*) is strictly regulated at both transcriptional and posttranscriptional levels. Efficient nitrogenase activity requires maintaining sufficient levels of *nif* mRNAs, yet the underlying mechanism is not fully understood due to its complexity. We have previously shown that a novel regulatory noncoding RNA (ncRNA), NfiS, optimizes nitrogen fixation through targeting *nifK* mRNA in Pseudomonas stutzeri A1501. Here, we report the identification and characterization of a second ncRNA inducible under nitrogen fixation conditions (nitrogen-free and microaerobic conditions), termed NfiR (for nitrogen fixation condition-inducible ncRNA), the expression of which is dependent on two global regulators, NtrC and Hfq. Comparative phenotypic and proteomic analyses of an *nfiR* mutant identify a role of NfiR in regulating the expression of nitrogenase genes. Further microscale thermophoresis and genetic complementation showed that an 11-nucleotide (nt) sequence in the stem-loop structure of NfiR (nucleotides 12 to 22) pairs with its counterpart in the coding region of *nifD* mRNA (nucleotides 1194 to 1207) by eight nucleotides. Significantly, deletion of *nfiR* caused a 60% reduction of nitrogenase activity, and the half-life of *nifD* mRNA was reduced from 20 min for the wild type to 15 min for the Δ*nfiR* mutant. With regard to nitrogenase activity and stability of the *nifD* and *nifK* transcripts, phenotypes were more severe for the double deletion mutant lacking *nfiR* and *nfiS*, suggesting that NfiR, in concert with NfiS, optimizes nitrogenase production at the posttranscriptional level.

**IMPORTANCE** Biological nitrogen fixation is an energy-expensive process requiring the hydrolysis of 16 ATPs. Consequently, the expression of *nif* genes is highly regulated at both transcriptional and posttranscriptional levels through complex regulatory networks. Global regulation involves a number of regulatory proteins, such as the *nif*-specific activator NifA and the global nitrogen regulator NtrC, as well as various regulatory ncRNAs. We show that the two P. stutzeri ncRNAs, namely NfiS and NfiR (for nitrogen fixation condition-inducible ncRNA), optimize nitrogen fixation and environmental stress responses. NfiS and NfiR respond differently to various environmental signals and differ in their secondary structures. In addition, the two ncRNAs target the mRNAs of *nifK* and *nifD*, respectively. Such ncRNA-based posttranscriptional regulation of nitrogenase expression might be an evolved survival strategy, particularly in nitrogen-limiting environments. This study not only highlights the significant roles of regulatory ncRNAs in the coordination and ﬁne tuning of various physiological processes but also provides a new paradigm for posttranscriptional regulation in nitrogen-fixing bacteria.

## Abstract (precis)

Biological nitrogen fixation is an energy-expensive process requiring the hydrolysis of 16 ATPs. Consequently, the expression of *nif* genes is highly regulated at both transcriptional and posttranscriptional levels through complex regulatory networks. Global regulation involves a number of regulatory proteins, such as the *nif*-specific activator NifA and the global nitrogen regulator NtrC, as well as various regulatory ncRNAs. We show that the two P. stutzeri ncRNAs, namely NfiS and NfiR (for nitrogen fixation condition-inducible ncRNA), optimize nitrogen fixation and environmental stress responses. NfiS and NfiR respond differently to various environmental signals and differ in their secondary structures. In addition, the two ncRNAs target the mRNAs of *nifK* and *nifD*, respectively. Such ncRNA-based posttranscriptional regulation of nitrogenase expression might be an evolved survival strategy, particularly in nitrogen-limiting environments. This study not only highlights the significant roles of regulatory ncRNAs in the coordination and ﬁne tuning of various physiological processes but also provides a new paradigm for posttranscriptional regulation in nitrogen-fixing bacteria.

## INTRODUCTION

Bacteria belonging to the genus Pseudomonas are found ubiquitously in many biotic and abiotic environments, including soil and water, as well as on the surfaces of plants, where nutrients are probably limited (1–5). *Pseudomonas* strains have evolved versatile metabolic capacity and complex regulatory networks for adaptation to nutrient-limited conditions (6–8). Global regulation involves a number of regulatory proteins, such as the sigma factor RpoN, the sigma factor RpoS, the global nitrogen regulator NtrC, and the noncoding RNA (ncRNA) chaperone Hfq (9–11). These regulatory proteins play key roles in global responses to environmental stresses.

In addition to regulatory proteins, various regulatory ncRNAs have been reported in pseudomonads, the majority of which appear to adjust bacterial physiology in response to environmental changes or nutrient stress conditions (12–14). It is particularly noteworthy that nitrogen stress-induced ncRNAs were verified as new players in nitrogen regulatory networks. NrsZ is the first nitrogen-regulated ncRNA reported in Pseudomonas aeruginosa, and it activates the production of rhamnolipid surfactants via posttranscriptional control of the rhamnolipid synthesis gene *rhlA* (15). Furthermore, several nitrogen stress-induced ncRNAs, the expression of which is activated by HetR, have been identified in the model filamentous nitrogen-fixing cyanobacterium Anabaena sp. strain PCC7120 (16). NsiR1 is the first known nitrogen stress-induced ncRNA present in a cyanobacterium, and it functions as an early marker of cell differentiation (17). Another nitrogen stress-induced ncRNA, NsiR4, is involved in nitrogen assimilation control in cyanobacteria by targeting glutamine synthetase (18). However, until very recently, ncRNAs had not been experimentally identified as regulators directly involved in nitrogen fixation. NfiS in Pseudomonas stutzeri A1501 represents the first ncRNA required for optimal expression of nitrogenase genes, and NfiS directly targets the *nifK* mRNA (19). In Methanosarcina mazei strain Go1, an ncRNA designated small RNA 154 (sRNA_154_) was found to play a central regulatory role in nitrogen metabolism via determining the expression of nitrogenase and glutamine synthetase by positively affecting transcript stabilities (20). Most recently, another archaeal ncRNA, sRNA_41_, was identified to regulate nitrogenase expression in an indirect manner by increasing levels of ACDS protein (acetyl coenzyme A-decarbonylase/synthase complexes) under nitrogen limitation (21).

Certain P. stutzeri strains possess a general nitrogen regulatory system (NtrBC and related genes) in the core genome; during evolution, they acquired the capacity to fix nitrogen via horizontal transfer of a 49-kb nitrogen fixation (*nif*) island (PST1302 to PST1359) that encoded a set of the proteins for synthesis, maturation, and functioning of nitrogenase, as well as two negative/positive regulatory proteins (NifL and NifA) for *nif* gene expression (1, 22, 23). Thus, the expression of *nif* genes in these strains is controlled by two regulatory systems of different evolutionary origins (24, 25). This global regulation includes a nitrogen regulator, NtrC, which is encoded in the core genome and is highly conserved among *Pseudomonas* species (9, 26). Under nitrogen fixation conditions, NtrC is phosphorylated, thereby causing transcriptional activation of *nifA* and carrying a positive regulator of the *nif* operons within the *nif* island (27). P. stutzeri strain A1501 is a diazotrophic proteobacterium originally isolated from the rice rhizosphere (28). It normally colonizes root surfaces but can also penetrate the root and grow endophytically. Genes for the synthesis, maturation, and functioning of nitrogenase are clustered in a 49-kb genomic island, suggesting that the nitrogen fixation property is acquired by lateral gene transfer from a diazotrophic ancestor (22). Although the importance of regulatory ncRNAs is generally recognized, their roles in fine-tuning expression of the horizontally acquired *nif* genes are poorly understood.

Here, we report the identification of a second regulatory ncRNA (termed NfiR for nitrogen fixation condition-inducible ncRNA), which is involved in the posttranscriptional control of the *nifD* gene. Our study began with transcriptome analysis of P. stutzeri A1501 for cells grown under nitrogen fixation and ammonium shock conditions. This analysis led to the identification of NfiR, whose expression is dependent on NtrC and Hfq and is significantly induced under nitrogen fixation conditions. Subsequent analyses demonstrated that NfiR is capable of binding to *nifD* transcripts *in vitro*, and this direct interaction can potentially affect the stability of the transcript. These data allow us to propose a model for the posttranscriptional control of the nitrogenase gene expression that involves two ncRNAs (NfiS and NfiR) in P. stutzeri A1501.

## RESULTS

### Transcriptome analysis of ncRNAs under nitrogen fixation conditions.

A global transcriptional profiling analysis was conducted with P. stutzeri A1501 to investigate the intracellular nitrogen stress responses, and the results are shown in Table S1. A total of 53 ncRNAs were detected under nitrogen fixation conditions, 17 of which were upregulated under nitrogen fixation conditions (nitrogen-free and microaerobic conditions) but were rapidly downregulated after 10 min of ammonium shock (Table S1). A BLASTN search of the 17 nitrogen-responsive ncRNA sequences against the GenBank database showed that (i) three ncRNAs are specific to strain A1501, without homologs in any other bacterial genomes, (ii) six ncRNAs are restricted to P. stutzeri, and (iii) eight ncRNAs are present in the genomes of other *Pseudomonas* species or bacteria. Notably, only four ncRNAs (NfiS, CrcZ, CrcY, and signal recognition particle bacterial RNA [SRP bact RNA]) have been functionally characterized, and there were no clues regarding the functions for the remaining 13 ncRNAs. This catalog of candidate regulatory ncRNAs will serve as an important reference point for comprehensive analyses of ncRNA regulation in P. stutzeri and other nitrogen-fixing bacteria.

**FIG 1:**
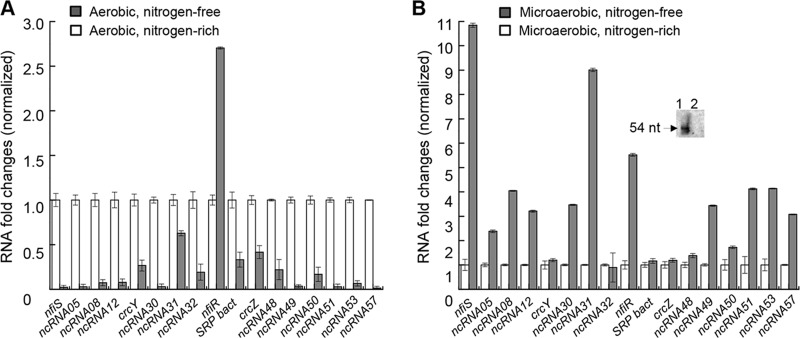
Quantitative RT-PCR analysis of the relative expression levels of the 17 ncRNAs under different growth conditions. (A) Nitrogen-free aerobic conditions (20% oxygen tension, black) versus nitrogen-sufficient aerobic conditions (6 mM NH_4_^+^ and 20% oxygen tension, white). (B) Nitrogen-free microaerobic conditions (0.5% oxygen tension, black) versus nitrogen-sufficient microaerobic conditions (6 mM NH_4_^+^ and 0.5% oxygen tension, white). Inset shows Northern blot detection of the NfiR RNA. Total RNA was extracted from wild-type strain A1501 under nitrogen-free microaerobic conditions (lane 1) and nitrogen excess conditions (lane 2). A1501 is able to fix nitrogen for its growth only under nitrogen-free microaerobic conditions. Data are the means and standard deviations of three independent experiments.

To gain insights into the potential roles of the 17 nitrogen-regulated ncRNAs in nitrogen stress responses, we monitored the gene transcription levels under both nitrogen fixation and non-nitrogen fixation conditions (Fig. 1). Under nitrogen-free and aerobic (20% oxygen tension) conditions, all ncRNAs were downregulated, with the exception of ncRNA34 (renamed NfiR), the expression of which was significantly upregulated compared to that under nitrogen-sufficient and aerobic conditions (Fig. 1A). In addition, almost all selected ncRNAs were induced under the nitrogen-free and microaerobic conditions appropriate for nitrogen fixation, compared with nitrogen-free, aerobic conditions, implying that they might be involved in the regulation of nitrogen fixation (Fig. 1B). Obviously, expression of NfiS shows the most dramatic increase (>10-fold), followed by that of ncRNA31 and then that of ncRNA34 (8.8- and 5.5-fold, respectively), under nitrogen fixation conditions. Since P. stutzeri A1501 was isolated from the rice rhizosphere, it is tempting to speculate that many ncRNAs are induced under nitrogen-free and microaerobic conditions to adapt to the nitrogen-poor and microaerobic rhizosphere environments. Further predictions of the interactions between the selected ncRNAs and *nif* gene mRNAs revealed that all selected ncRNAs have no binding sites for *nif* gene mRNAs, except for NfiS, with an experimentally confirmed binding site for *nifK* mRNA (19), and ncRNA34, with a putative binding site for *nifD* mRNA (Fig. S1A). Hence, ncRNA34 was redesignated NfiR (nitrogen fixation condition-inducible ncRNA).

**FIG 2:**
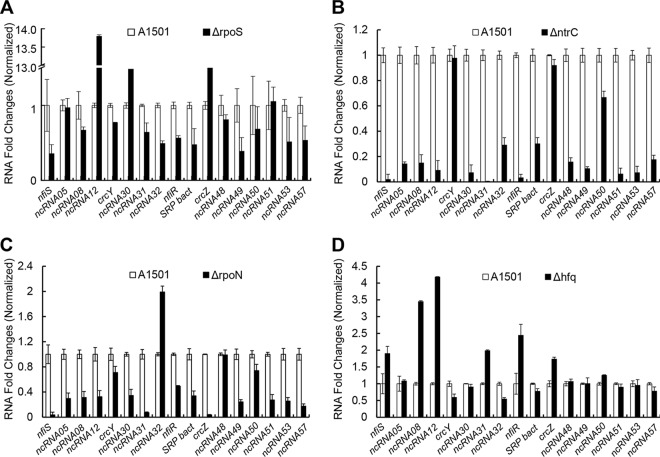
Quantitative RT-PCR analysis of relative expression levels of the 17 ncRNAs in wild-type and mutant backgrounds. (A) P. stutzeri A1501 (wild type, white) versus the *rpoS* mutant (*ΔrpoS*, black). (B) P. stutzeri A1501 (wild type, white) versus the *ntrC* mutant (*ΔntrC*, black). (C) P. stutzeri A1501 (wild type, white) versus the *rpoN* mutant (Δ*rpoN*, black). (D) P. stutzeri A1501 (wild type, white) versus the *hfq* mutant (*Δhfq*, black). Measurements were normalized to the wild-type values, and fold differences are plotted. Data are the means and standard deviations of three independent experiments.

Expression of many ncRNAs is associated with environmental stresses and is regulated by various regulatory proteins, such as the sigma factor RpoN, the global nitrogen activator NtrC, the sigma factor RpoS, and the RNA chaperone Hfq (16, 18, 19). Thus, we compared the expression levels of the 17 ncRNAs between wild-type A1501 and each of four isogenic mutants devoid of RpoN, NtrC, RpoS, or Hfq (Fig. 2). Deletion of *rpoS* caused decreased expression of 12 ncRNAs and increased expression of ncRNA12 (>13.8-fold), an ncRNA of unknown function specific to A1501, and CrcZ (>2.7-fold), a protein-binding ncRNA acting as global regulator of carbon catabolite repression (Fig. 2A) (29). Most of the ncRNAs were downregulated in the *ntrC* or *rpoN* deletion backgrounds (Fig. 2B and C), except for ncRNA32, whose predicted target is the *rnfA* gene encoding the subunit A of the electron transport complex and is probably involved in nitrogen fixation. Of particular note is that the transcription of three ncRNAs (NfiS, NfiR, and ncRNA31, whose predicted target is the *hesB* gene encoding Fe-S cluster assembly protein probably involved in nitrogen fixation) was abolished in the *ntrC* deletion background (Fig. 2B), suggesting that their expressions are transcriptionally activated by NtrC. Furthermore, Hfq appears to be a pleiotropic regulator of gene expression, as a loss-of-function mutation led to up- or downregulation of the selected ncRNAs, such as *nfiR* and *nfiS*, whose expression levels were upregulated by approximately 2- and 3-fold, respectively (Fig. 2D). Taken together, transcriptome sequencing (RNA-seq) analysis of A1501 grown under nitrogen fixation conditions identified 17 nitrogen-regulated ncRNAs that respond differently to nitrogen and oxygen signals and differ in transcriptional regulation, presumably constituting a complex regulatory network to integrate nitrogen fixation with global cellular physiology. Among these ncRNAs, in addition to a previously described NfiS, NfiR is the only ncRNA that has a putative binding site with nitrogenase gene mRNA, implying its potential involvement in the posttranscriptional control of nitrogen fixation.

### Phenotypic characterization of a mutant lacking the *nfiR* gene.

The *nfiR* gene, located in the intergenic region between PST2408 (a hypothetical protein) and PST2409 (a putative assimilatory nitrite reductase, NasA) (Fig. S1B and C), is predicted to encode a 54-nucleotide (nt) transcript (Fig. 1B), representing the smallest ncRNA that has been functionally characterized thus far in *Pseudomonas*. Furthermore, we detected several *nfiR* homologs, including an experimentally characterized P. aeruginosa sRNA P11 (30) and a predicted Azotobacter vinelandii sRNA11, but their functions are unknown. In addition, we did not identify homologs in any other bacterial species, suggesting that *nfiR* is specific to *Pseudomonas*.

**FIG 3:**
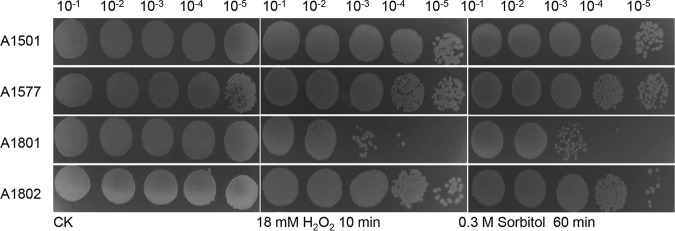
Survival phenotype plate assay with oxidative or osmotic stress. Serial 10-fold dilutions of OD-standardized cultures were spotted on LB plates after exposure to 18 mM H_2_O_2_ or 0.3 M sorbitol. A1501, wild type; A1577, A1501 containing pLAFR3; A1801, Δ*nfiR* mutant; A1802, complemented strain; CK, untreated culture control.

In nitrogen-fixing bacteria, certain nitrogen-regulated ncRNAs were verified as regulatory players in coupling between nitrogen metabolism and stress responses (14, 15). To determine whether *nfiR* is involved in the regulation of nitrogen fixation and stress resistances, we constructed an *nfiR*-knockout mutant, A1801 (Δ*nfiR*), and a complementary strain, A1802, corresponding to A1801 harboring a plasmid expressing the wild-type *nfiR* gene, and we compared their growth under stress conditions by the addition of sorbitol or H_2_O_2_ with the growth of wild-type strain A1501. As shown in Fig. 3, both A1801 and A1802 displayed growth rates similar to that of the wild-type strain in Luria-Bertani (LB) medium, indicating that deletion of the *nfiR* gene had no effect on bacterial survival under normal growth conditions. Furthermore, we found that in the presence of 18 mM H_2_O_2_ or 0.3 M sorbitol, the *nfiR* mutant displayed significantly impaired growth, but the complementary strain recovered the growth capacity to the wild-type level under the same treatment, implying that NfiR might play an important role in the response to oxidative or osmotic stress. To this end, we compared global protein expression changes between wild-type A1501 and its derived *nfiR* mutant (Fig. S2). A total of 258 spots with 1.5-fold or greater differences in fluorescence intensity were identified, and the identities of 123 spots were successfully determined using matrix-assisted laser desorption ionization–time of flight (MALDI-TOF) mass spectrometry (Table S2). These include three cell mobility-related proteins undetectable in the *nfiR* mutant and several stress response proteins undetectable in the wild-type strain. In addition, a glutathione *S*-transferase (GST; PST3481) was found to be 60.0-fold more abundant in the *nfiR* mutant than in the wild type. GST participates in protecting cells against damage due to oxidative stress in proteobacteria (31); however, its functions in Pseudomonas stutzeri still remain to be elucidated. Unexpectedly, GST seems to be substantially derepressed in the *nfiR* mutant, although it is not yet known whether NfiR regulates the protein directly or acts indirectly to alter its synthesis. We anticipate that NfiR mutation causes global changes in gene expression that may lead to more complex effects on oxidative stress response than previously thought.

**FIG 4:**
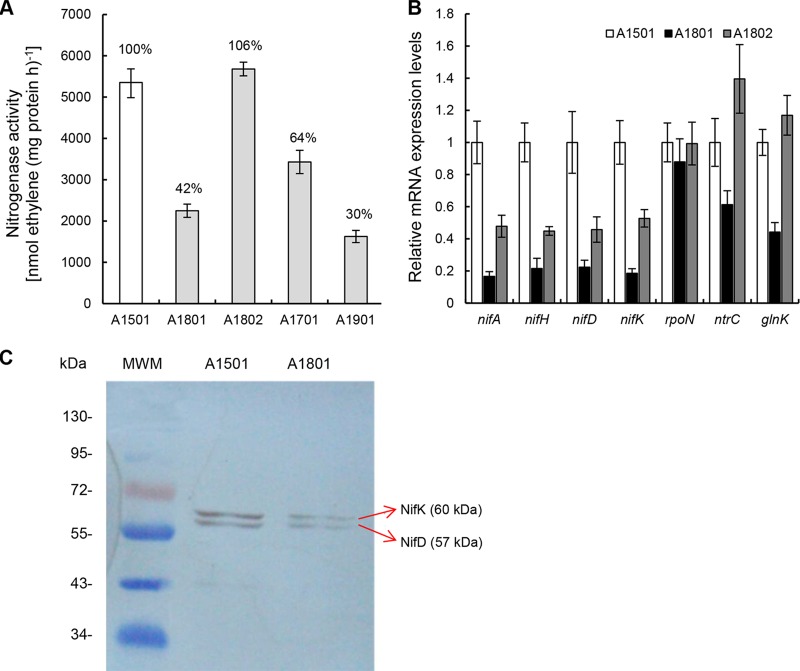
NfiR is required in conjunction with NfiS for optimal nitrogenase activity. (A) Nitrogenase activity in wild-type A1501; three isogenic mutants, A1801 (Δ*nfiR*), A1701 (Δ*nfiS*), and A1901 (Δ*nfiR* Δ*nfiS*); and the complemented strain A1802 (A1801 containing pnfiR-wt). The percent changes in nitrogenase activity from the wild-type value are also indicated. (B) The effect of *nfiR* deletion on the expression of *nifHDK* genes and their regulators. Relative levels of transcripts are presented as mean values ± standard deviations (SDs) calculated from three sets of independent experiments and normalized to levels in the wild-type strain. (C) Western blot analysis of the nitrogenase MoFe protein NifD and NifK polypeptides. Lane 1, molecular weight marker (MWM); lane 2, A1501, wild type; lane 3, A1801, Δ*nfiR*. NifD and NifK polypeptides are indicated by the arrows.

Furthermore, we found that deletion of *nfiR* resulted in a significant reduction of nitrogenase activity by more than half, and the defect was restored by the introduction of a single copy of *nfiR* (Fig. 4A). The results of quantitative real-time PCR (qRT-PCR) showed that the expression levels of *nifA*, *nifH*, *nifD*, *nifK*, and their regulatory genes *rpoN*, *ntrC*, and *glnK* were decreased to various extents in the *nfiR* mutant compared with the wild type, whereas these inductions were fully or partially restored to wild-type levels by the complementation plasmid with a wild-type *nfiR* gene (Fig. 4B). Furthermore, the Western blot results showed that nitrogenase MoFe protein polypeptides (NifD and NifK) were produced at lower levels in the *nfiR* mutant background than in the wild type (Fig. 4C). These data consistently indicate that NfiR is a positive regulator required for maximal expression of nitrogenase. These findings are consistent with results from the proteomics analysis (Fig. S2). Proteins associated with nitrogen metabolism and nitrogen fixation, such as the glutamine synthetase GlnA, Fe-S cluster assembly protein NifU, and MoFe protein alpha subunit NifD, were downregulated in the *nfiR* mutant (Table S2). Together, our data suggest a role for NfiR in regulating the expression of proteins involved in nitrogen fixation, as well as in osmotic and oxidative resistances.

### Involvement of Hfq and NtrC in the regulation of NfiR expression.

The ncRNA chaperone Hfq is usually required for the function and/or stability of most base-pairing ncRNAs (32). Previous studies have also shown that although expression of the *nfiS* gene was upregulated in the *hfq* deletion, the half-life of the NfiS transcript in the wild-type strain was more than twice that of the *hfq* mutant (19). Similarly, the *hfq* deletion also resulted in upregulation of *nfiR* expression (Fig. 2D). When wild-type cells were grown to the mid-exponential phase and treated with rifampin, *nfiR* mRNA had a half-life of approximately 4 min. In contrast, the *hfq* mutant had a half-life of 2 min (Fig. S3). In addition, deletion of *hfq* resulted in decreased expression of the assayed *nif* and *nif*-related genes (Fig. S3B), consistent with the previous results that inactivation of *hfq* caused a significant decrease in nitrogenase activity (19). Our data indicate that Hfq enhances the stability of both ncRNAs while it functions in a global manner to control their transcriptional activities via unknown mechanisms.

**FIG 5:**
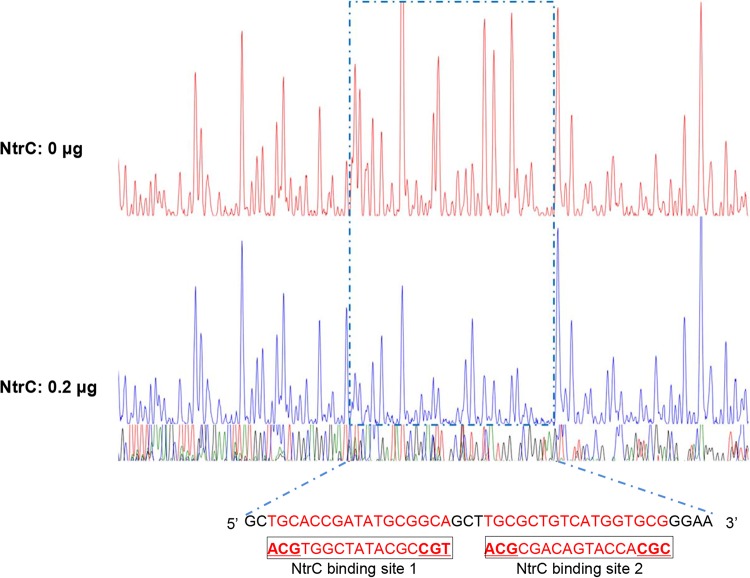
Binding of purified NtrC protein with *nfiR* promoter DNA. DNase I footprinting analysis of the *nfiR* promoter probe (500 ng) without addition of purified NtrC protein (upper panel) or with purified NtrC protein added at 0.2 μg (lower panel). The NtrC-protected region is indicated by a blue dotted box, with the nucleotide sequence shown at the bottom. The predicted NtrC-binding sites are marked by boxes.

Interrogation of the NfiR promoter DNA indicated the presence of two NtrC-binding sites, which contain the highly conserved TGC and GCA elements with an 11-nucleotide spacing (Fig. 5). Moreover, *nfiR* expression was abolished in the Δ*ntrC* background (Fig. 2B). Evidence available thus far has strongly suggested that NtrC activates NfiR expression in a direct manner. To this end, we performed a DNase I protection footprint sequencing assay to determine the NtrC-protected region using a 264-bp *nfiR* promoter DNA fragment as the probe. The results indicate that NtrC was capable of protecting a 43-bp DNA region containing the predicted two putative NtrC-binding sites (Fig. 5). Next, we performed 5′ rapid amplification of cDNA ends (5′ RACE) to identify the transcription start site of the *nfiR* gene. Contrary to our expectation, NfiR transcription did not initiate from the 12 position downstream of the predicted σ^54^-binding site; instead, it started from a cytosine residue located 29 bp downstream of the predicted σ^54^-binding site (Fig. S1A). Curiously, *nfiR* expression was reduced (but not abolished) in the genetic background of Δ*rpoN* (Fig. 2C). Together, these data show that NfiR expression is subject to complex regulation at both the transcriptional and posttranscriptional levels, which involves both Hfq and NtrC.

### Examining the molecular interactions between NfiR and the *nifD* mRNA.

**FIG 6:**
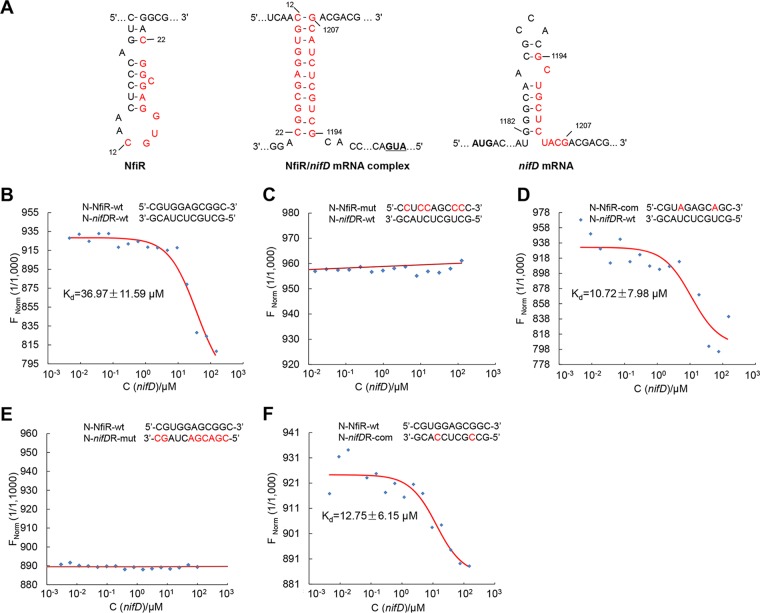
Molecular interactions between NfiR and *nifD* mRNA. (A) Schematic representation of the predicted base-pairing complex formation (middle) between the NfiR stem-loop (left) and the complementary sequence of *nifD* mRNA (right). Pairing nucleotides are shown in red. (B to F) Determination of the affinity of NfiR binding to *nifD* mRNA by microscale thermophoresis. The concentration of labeled N-NfiR was constant, whereas the concentrations of the unlabeled binding partner N-*nifD*R molecules varied from 10 nM to 300 μM. Point mutations introduced into synthesized oligonucleotides are shown in red. N, synthesized oligonucleotide; mut, mutant allele; com, complementary mutation; *nifD*R, *nifD* mRNA; wt, wild type.

*In silico* analysis using RNAalifold (33) revealed the presence of an 11-nt sequence in the stem-loop structure of NfiR (nucleotides 12 to 22) pairing with its counterpart in the coding region of *nifD* mRNA (nucleotides 1194 to 1207) by eight nucleotides (Fig. 6A). To validate the predicted interaction, we first synthesized two 30-nt single-stranded RNA (ssRNA) oligonucleotides containing the wild-type base-pairing sequence of NfiR or *nifD* mRNA, designated N-NfiR-wt and N-*nifD*R-wt, plus another two containing an absolute mismatch mutation (N-NfiR-mut) and full complementary mutations (N-NfiR-com). The results of microscale thermophoresis (MST) showed that N-NfiR-wt (82% match level) was capable of binding with N-*nifD*R-wt, exhibiting a dissociation constant of 36.97 ± 11.59 μM (Fig. 6B). Significantly, no binding signal was detected with the N-NfiR-mut molecule carrying five substitutions in the base-pairing sequence of NfiR (Fig. 6C). Next, we measured the binding affinity using N-NfiR-com (100% match level). The complementary mutation resulted in enhanced binding affinity to N-*nifD*R-wt compared with the wild-type situation, showing a dissociation constant of 10.72 ± 7.98 μM (Fig. 6D). Finally, the effect of *nifD* mRNA mutations in the 11-nt sequence (1194 to 1207 nt) on base pairing with NfiR was investigated using 30-nt ssRNA oligomers containing a mismatch mutation (N-*nifD*R-mut) and a complementary mutation (N-*nifD*R-com). The results indicate that N-*nifD*R-mut (100% mismatch) showed no binding affinity for NfiR (Fig. 6E), whereas N-*nifD*R-com (perfect match) displayed a stronger interaction with NfiR than N-*nifD*R-wt (Fig. 6F). Together, these data strongly indicate that NfiR is capable of interacting with the *nifD* mRNA *in vitro*.

To further confirm functions of the predicted NfiR sequence *in vivo*, we measured nitrogenase activities of the Δ*nfiR* mutant complemented with either wild-type *nfiR* or each of the four mutant *nfiR* alleles carrying one or more guanine-to-cytosine substitutions in the base-pairing region (Table 1). To this end, DNA fragments of wild-type and mutant *nfiR* were individually cloned into the plasmid vector pLAFR3, generating five complementation plasmids, pNfiR-wt, pNfiR-mut1, pNfiR-mut2, pNfiR-mut3 and pNfiR-mut4. The resultant plasmids were then transformed into the *nfiR* mutant (strain A1801). As shown in Table 1, the nitrogenase activity of the NfiR mutant A1801 was almost fully complemented by the wild-type *nfiR* allele but was only partially complemented by the mutated *nfiR* genes. The mutated *nfiR* gene NfiR-mut1, with one nucleotide substitution (G_13_→C), restored 91% of wild-type nitrogenase activity, whereas NfiR-mut4, with five nucleotide substitutions, restored only 19%. Most interestingly, the nitrogenase activity of A1805 carrying NfiR-mut3, with two nucleotide substitutions (G_15_G_16_→CC), was restored to 20% of the wild-type level, much lower than that of A1804 carrying NfiR-mut2, with two nucleotide substitutions (G_20_G_21_→CC), but almost equal to that of A1806 carrying NfiR-mut4, with five nucleotide substitutions (G_13_→C, G_15_G_16_→CC, and G_20_G_21_→CC), highlighting the importance of the two guanines at positions 15 and 16 in NfiR regulatory function. These results strongly indicate that the 11-nt sequence within its stem-loop structure is functionally required for NfiR to regulate optimal nitrogen fixation.

**TABLE 1:** Nitrogenase activities of A1801 (Δ*nfiR*) carrying the wild-type *nfiR* gene or each of the four mutated *nfiR* genes

Strain	Complementation plasmid^*a*^	Sequence with point mutation(s)^*b*^	Match level (%)^*c*^	Nitrogenase activity^*d*^	% change^*e*^
A1501			82	5,354.79 ± 327	100
A1801				2,249.01 ± 160	42
A1802	pNfiR-wt	5′-CGUGGAGCGGC-3′	82	5,679.97 ± 166	106
A1803	pNfiR-mut1	5′-C**C**UGGAGCGGC-3′	73	4,855.23 ± 167	91
A1804	pNfiR-mut2	5′-CGUGGAGC**CC**C-3′	73	3,596.57 ± 169	67
A1805	pNfiR-mut3	5′-CGU**CC**AGCGGC-3′	73	1,078.94 ± 76	20
A1806	pNfiR-mut4	5′-C**C**U**CC**AGC**CC**C-3′	55	1,018.99 ± 60	19

a The intact wild-type NfiR gene and four mutated *nfiR* genes with one or more nucleotide substitutions were cloned into plasmid pLAFR3, and the resulting complementation plasmids were introduced into A1801 (Δ*nfiR*), generating the five complementation strains.

b Point mutations introduced into synthesized oligonucleotides are shown in boldface.

c The match level (%) of sequences base pairing between NfiR and *nifD* mRNA.

d Nitrogenase activity is expressed as nmol of ethylene per hour per milligram protein.

e The percent change (%) in nitrogenase activity of the complementation strains is based on comparison with activity of the wild-type A1501.

### Both NfiR and NfiS are required for optimal expression of nitrogenase genes.

**FIG 7:**
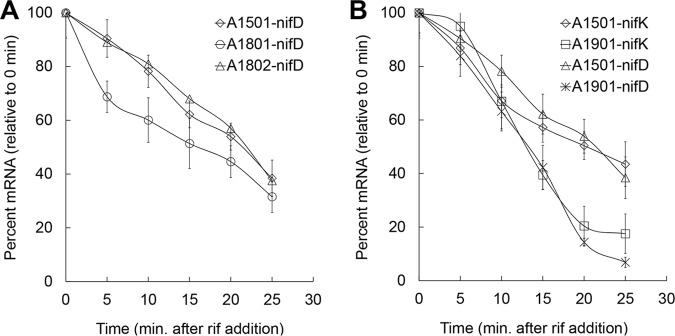
Effects of *nfiR* and/or *nfiS* deletion on stability of the *nifD* and *nifK* transcripts. (A) Half-lives of the *nifD* transcript in wild-type A1501, A1801 (Δ*nfiR*), and A1802 (complementation strain). (B) Half-lives of the *nifD* and *nifK* transcripts in wild-type A1501 and the double deletion mutant A1901. Rifampin (rif) (400 μg/ml) was added at time 0. At the times indicated (0, 5, 10, 15, 20, and 25 min) an equal volume of cold medium was added to bring the temperature immediately to 4°C. RNA was extracted, followed by quantitative real-time PCR (qRT-PCR). Data are the means and standard deviations of three independent experiments.

Base-pairing interactions between ncRNAs and their target mRNAs often regulate the stability of the mRNA transcripts (34). We have previously shown that NfiS increases the half-life of *nifK* mRNA (19). To test whether this function holds for NfiR, we measured the half-life of *nifD* mRNA in wild-type A1501, *nfiR* mutant (strain A1801), and the complementation mutant A1802 (Fig. 7A). qRT-PCR was performed for cells grown under nitrogen fixation conditions. Rifampin was added to inhibit RNA synthesis. The results showed that the *nifD* mRNA half-life was 20 min after rifampin had been added to wild-type cells, and it was reduced to 15 min in the *nfiR* mutant. The *nifD* transcript stability of the *nfiR* mutant was restored with the introduction of wild-type *nfiR* (Fig. 7A). Consistent with our expectation, the double deletion mutant (A1901) lacking *nfiR* and *nfiS* had reduced half-lives for both *nifD* and *nifK* mRNAs compared with the wild type (Fig. 7B). Intriguingly, a 70% reduction was observed for the *nfiR* and *nfiS* double deletion mutant, whereas deletion of either *nfiS* or *nfiR* caused a 40 or 60% reduction of the nitrogenase activity, respectively (Fig. 4A). Together, the data from the nitrogenase activity and mRNA stability assays strongly suggest that both NfiR and NfiS optimize nitrogenase activity in a cooperative manner via determining the stabilities of the nitrogenase gene mRNAs.

## DISCUSSION

Biological nitrogen fixation is an ancient trait dating back to more than 3 billion years ago (35, 36). It is an energy-expensive process, requiring large amounts of both reducing power and high-energy phosphate (ATP). To adapt to diverse environments and to avoid energy waste, nitrogen-fixing bacteria have evolved various regulatory mechanisms to control the transcription of *nif* genes and the synthesis and activity of nitrogenases in response to ammonium availability and other environmental factors (37–39). This global regulation occurs at transcriptional and posttranscriptional levels, including a classical nitrogen regulatory cascade, an ADP-ribosyl-transferase/glycohydrolase (DraT/DraG) system, and a recently identified complex network of ncRNAs. The RpoN-NtrC-NifA regulatory cascade is a major and best-studied regulatory mechanism for nitrogen fixation at the transcriptional level (27, 40). In addition, nitrogenase activity can be modulated by ADP ribosylation of NifH, the so-called “switch-off” effect. The best-characterized mechanism of switch-off involves the ADP ribosylation of NifH by the DraT/DraG system in response to the addition of excess ammonium (41). Although A1501 does not carry *draTG* (22), nitrogenase switching off of this strain has been reported (26), suggesting an inactivation mechanism different from that reported in other nitrogen-fixing bacteria (35). Currently, the ncRNA-based posttranscriptional regulatory mechanisms of nitrogen fixation have not been fully elucidated.

In this study, we conducted a global transcriptional profiling analysis and identified 17 nitrogen-regulated ncRNAs. Further analysis revealed that these ncRNAs respond differently to nitrogen and oxygen signals and differ in transcriptional regulation (Fig. 1 and 2), presumably constituting a complex regulatory network to integrate nitrogen fixation with global cellular physiology. Furthermore, we employed the computational software sRNATarget to predict their potential targets and found that all selected ncRNAs have no binding sites for *nif* gene mRNAs, except for NfiS and NfiR. In the case of nitrogen-fixing P. stutzeri, NfiS is identified as the first regulatory ncRNA that regulates optimal nitrogen fixation via direct base pairing with *nifK* mRNA (19). Here, we identified the second P. stutzeri ncRNA, NfiR, which is potentially involved in base-pairing interaction with the *nifD* transcript. Both ncRNAs are conserved in P. stutzeri and play global regulatory roles in environmental stress responses in addition to their roles in nitrogenase gene expression. Our findings strongly suggest that in P. stutzeri A1501, NfiR and NfiS act as new riboregulators to integrate the horizontally acquired *nif* island into host global networks. The precise molecular mechanisms for modulating NfiS and NfiR activities are not known. Although both ncRNAs are expressed at elevated levels under nitrogen fixation conditions, they respond in the opposite way under nitrogen-free conditions, namely, NfiS is downregulated (19), whereas NfiR is upregulated (Fig. 1A). Furthermore, NfiS and NfiR differ in their promoter sequences and transcriptional regulations. We previously identified a conserved σ^54^-binding site on the *nfiS* promoter (19). NfiS expression is abolished in the *rpoN* background, thereby confirming that it is a σ^54^-dependent ncRNA (19). No NtrC-binding site was identified in the *nfiS* promoter. In contrast, we characterized the promoter sequence of *nfiR* and identified a conserved NtrC-binding site (Fig. S1A). The DNase I footprinting assay *in vitro* confirmed that NtrC binds specifically to the *nfiR* promoter (Fig. 5).

Nitrogen fixation is significantly affected by environmental stresses, such as nitrogen starvation or oxidative or osmotic stresses encountered by associative nitrogen-fixing bacteria in the rhizosphere. Consequently, certain nitrogen-regulated ncRNAs were verified as regulatory players in coupling between nitrogen fixation and the stress response (14, 15). The primary function of NfiS is related to osmotic and oxidative stress responses, but it was later recruited and paired with *nifK* mRNA as a new riboregulator when ancestral P. stutzeri acquired a nitrogen fixation island through horizontal gene transfer (19). In this study, we found that NfiR is functionally related to osmotic and oxidative stress responses and that it was most likely recruited by *nifD* mRNA in a manner similar to NfiS. Furthermore, we confirmed that both NfiR and NfiS were upregulated by various transcriptional or posttranscriptional regulators, such as RpoS, NtrC, RpoN, and Hfq (Fig. 2), suggesting that the modulation of many cellular functions by protein regulators could be associated with the two ncRNAs. Such ncRNA-based regulation might be an evolved survival strategy, particularly in nitrogen-limiting environments.

NtrC is a response regulator with a σ^54^-interacting domain, which requires functional σ^54^ for its activities. Transcription initiation of σ^54^-dependent promoters usually begins 12 nt downstream of the σ^54^ binding site, which is followed closely by the translation initiation site. It is thus surprising to find that *nfiR* transcription started from a position 29 bp downstream of the predicted σ^54^-binding site (Fig. S1A). However, it is still possible that NtrC directly activates *nfiR* transcription in the conventional σ^54^-dependent manner; the primary product of NfiR is then subject to RNA processing at the 5′ end, producing a 54-nt ncRNA. A similar phenomenon has previously been reported in Pseudomonas putida with CrcY, an ncRNA involved in carbon catabolite repression (29). Alternatively, there may be some yet-unknown transcription factors involved in NtrC/σ^54^-mediated gene regulation that cause a shift of the transcriptional start site. It is also possible that NtrC produces additional effects on NfiR expression via a yet-unknown transcription factor(s), the activities of which are independent of σ^54^. This possibility would explain the observation that *nfiR* transcription was abolished in the Δ*ntrC* background, while it was reduced only by half in the Δ*rpoN* mutant. Nevertheless, NfiR expression is subject to complex regulation, and the precise roles of NtrC and σ^54^ require further investigation.

Results of microscale thermophoresis and genetic complementation strongly suggest that *nifD* mRNA is a direct target of NfiR. However, given the global effects associated with *nfiR* deletion, it is reasonable to speculate that NfiR targets multiple mRNAs, particularly those in the σ^54^ regulon. The expression levels of *rpoN* (the gene encoding σ^54^ in *Pseudomonas*) and other genes under the control of σ^54^, e.g., *nifA*, *nifD*, *nifK*, *ntrC*, and *glnK*, were decreased in the *nfiR* strain (Fig. 4B), indicating that their transcriptional activities were controlled in an ncRNA-dependent manner. Furthermore, proteomic analysis of the Δ*nfiR* mutant also identified a subset of differentially expressed proteins whose expression profiles are σ^54^ dependent (Table S2). Hence, it would make sense that σ^54^ is one of the primary targets for NfiR. However, no putative NfiR-binding sites were identified in the *rpoN* locus. Thus, the observed phenotypic effects related to σ^54^ function are likely mediated by NfiR directly targeting a regulator in the σ^54^ regulon.

Efficient nitrogenase activity greatly relies on the sufficient accumulation of *nif* mRNAs. Some diazotrophs can accumulate nitrogenase at levels of as much as 10% of the total soluble proteins within the cells (42), which would require relatively higher levels of *nifHDK* mRNAs than of mRNA specifying other proteins. Thus, nitrogen-fixing cells must have a robust capacity to produce *nifHDK* mRNAs at a level sufficient to sustain maximal nitrogenase activity. Such a cellular physiological requirement is consistent with the proposed functional roles of the P. stutzeri NfiS/NfiR and the *M. mazei* sRNA_154_ ncRNAs in enhancing the stability of their target mRNAs. It is interesting to note that sRNA_154_ was shown to be directly involved in regulating nitrogen fixation in Methanosarcina mazei (20). This ncRNA specifically targets the *nifH* mRNA. Despite originating from phylogenetically distinct organisms, sRNA_154_ possesses similar modes of action as NfiS and NfiR. Considering the high levels of conservation of the *nifHDK* genes among nitrogen-fixing bacteria, it is reasonable to speculate that P. stutzeri A1501 may carry a third as-yet-unidentified ncRNA that specifically targets the *nifH* transcript, as previously described for sRNA_154_ (20). Involvement of three ncRNAs targeting each of the three nitrogenase components (NifH, NifD, and NifK) would certainly strengthen the regulation of nitrogen fixation and may also help prevent unbalanced synthesis of the three proteins. More importantly, it would allow integration of additional environmental signals into the precise control of nitrogenase synthesis. In conclusion, our work provides evidence that two Hfq-dependent P. stutzeri ncRNAs, namely NfiS and NfiR, optimize nitrogen fixation and abiotic stress responses. In addition, the two ncRNAs target the mRNAs of *nifK* and *nifD*, respectively, increasing the half-life of the transcripts and, consequently, nitrogenase activity. This study not only highlights the significant roles of regulatory ncRNAs in the coordination and ﬁne tuning of various physiological processes but also provides a new paradigm for posttranscriptional regulation in nitrogen-fixing bacteria.

## MATERIALS AND METHODS

### Bacterial strains, media, and growth conditions.

A summary of bacterial strains used in this study is provided in Table 2. P. stutzeri strains were grown at 30°C in Luria-Bertani (LB) medium or in modified minimal lactate-containing medium (medium K) (26). When necessary, spectinomycin (Spc), kanamycin (Km), and chloramphenicol (Cm) were added at final concentrations of 34 μg/ml, 50 μg/ml, and 40 μg/ml, respectively.

**TABLE 2:** Strains and plasmids used in this study

Strain/plasmid	Relevant characteristic(s)	Source or reference
P. stutzeri strains		
A1501	Wild type, Chinese Culture Collection CGMCC 0351	28
A1550	*rpoN*-Cm deletion mutant, Cm^r^	26
A1565	*ntrC*-Cm deletion mutant, Cm^r^	49
A1507	*rpoS* deletion mutant; the *rpoS* gene was knocked out by homologous suicide plasmid integration using pK18*mobsacB* as the vector	Lab collection
A1521	*hfq* nonpolar insertion mutant, Km^r^	19
A1701	*nfiS*-Spc deletion mutant strain, Spc^r^	19
A1801	*nfiR*-Cm deletion mutant strain, Cm^r^	This study
A1901	*nfiR nfiS* double deletion mutant strain, Cm^r^ and Spc^r^	This study
A1802	A1801 containing *pnfiR*-wt, Cm^r^ and Tc^r^	This study
A1803	A1801 containing *pnfiR*-mut1, Cm^r^ and Tc^r^	This study
A1804	A1801 containing *pnfiR*-mut2, Cm^r^ and Tc^r^	This study
A1805	A1801 containing *pnfiR*-mut3, Cm^r^ and Tc^r^	This study
A1806	A1801 containing *pnfiR*-mut4, Cm^r^ and Tc^r^	This study
A1577	A1501 containing pLAFR3, Tc^r^	This study
Plasmids		
pK18mobsacB	Allelic exchange vector, Km^r^	44
pLAFR3	Mobilizable vector, Tc^r^	50
pRK2013	Helper plasmid for conjugation into P. stutzeri A1501, Km^r^	51
pKatCAT5	Source of chloramphenicol resistance cassette, Cm^r^	Lab collection
pMD18-T	2.96-kb cloning vector, Amp^r^	TaKaRa
pJET1.2	2.97-kb cloning vector, Amp^r^	TaKaRa
pK18/delR	pK18*mobsacB* derivative carrying a BamHI/HindIII fragment for homologous recombination, Cm^r^ and Km^r^	This study
pK18/delS	pK18*mobsacB* derivative carrying a BamHI/HindIII fragment for homologous recombination, Spc^r^, Km^r^	19
pNfiR-wt	pLAFR3 derivative carrying the wild-type *nfiR* gene under the control of its endogenous promoter, Tc^r^	This study
pNfiR-mut1	pLAFR3 derivative carrying a mutated *nfiS* gene with a substitution of one nucleotide within the pairing site region, Tc^r^	This study
pNfiR-mut2	pLAFR3 derivative carrying a mutated *nfiS* gene with a substitution of two nucleotides within the pairing site region, Tc^r^	This study
pNfiR-mut3	pLAFR3 derivative carrying a mutated *nfiS* gene with a substitution of two nucleotides within the pairing site region, Tc^r^	This study
pNfiR-mut4	pLAFR3 derivative carrying a mutated *nfiS* gene with a substitution of five nucleotides within the pairing site region, Tc^r^	This study

### Total RNA-seq and ncRNA prediction.

Strain A1501 was cultured for 5 h under nitrogen fixation and ammonium shock conditions as described previously (24, 25). RNA was extracted using TRIzol LS reagent (Invitrogen, USA) following the manufacturer’s instructions. Host-cell RNA was depleted using a MICROB*Enrich* kit (Ambion, USA), and bacterial 23S and 16S rRNAs were subsequently depleted with a MICROB*Express* bacterial mRNA enrichment kit (Ambion, USA). Total RNA-seq libraries were then constructed and sequenced using an Illumina HiSeq 2500 instrument with the paired-end method, and ncRNAs were predicted based on the mapping of read pairs by Tianjin Biochip Corporation (Tianjin, China).

### Growth conditions for transcriptional expression assay.

To test the expression levels of the selected 17 ncRNAs, cells of wild-type and mutant strains were grown in medium K and harvested at the early exponential phase. Subsequently, the cells were washed once with fresh medium K and used to start experiments at a final optical density at 600 nm (OD_600_) of 0.5 under the following four growth conditions: nitrogen starvation conditions (medium K, nitrogen-free, and aerobic conditions), nitrogen fixation conditions (medium K, nitrogen-free, and 0.5% oxygen tension), oxygen limitation conditions (medium K, nitrogen sufficient, and 0.5% oxygen tension), and normal conditions (medium K, nitrogen sufficient, and aerobic conditions). After incubation for 0.5 h at 30°C, cells were harvested and centrifuged for 10 min at 4°C, and pellets were quick-frozen in liquid nitrogen and stored at −80°C until ready for quantitative real-time PCR (qRT-PCR) assays.

### RNA isolation for qRT-PCR.

Total RNA was isolated from bacteria cultured under the described conditions using the SV total RNA isolation system (Promega, Madison, WI) according to the manufacturer’s instructions. Total RNA was quantified using microspectrophotometry (NanoDrop Technologies, Inc.). RNA integrity was measured using an Agilent 2100 Bioanalyzer (Agilent Technologies, Inc.). RNA samples with RNA integrity numbers (RINs) above 7.0 and threshold cycle (*C_T_*) values above 32 were used for qRT-PCR.

### Quantitative real-time PCR.

Expression levels of selected genes were determined by qRT-PCR with a Power SYBR green PCR master mix using an ABI Prism 7500 sequence detection system (Applied Biosystems, USA) according to the manufacturer’s instructions. Primers were designed based on sequences of selected genes, which were imported into OligoPerfect (Invitrogen), a primer design software program designed to generate primer pairs suitable for real-time PCR. Primers used for qRT-PCR are listed in Table 3. All qRT-PCRs were performed in triplicate using a 25-ml mixture containing cDNA (5 ml of a one-fifth dilution), 1× brilliant SYBR green quantitative PCR master mixture (Stratagene), and approximately 5 pmol of each primer. Amplification and detection of specific products were performed using the following procedure: 95°C for 10 min, followed by 40 cycles of 95°C for 30 s, 55°C for 1 min, and 72°C for 30 s and then a dissociation curve analysis. The 16S rRNA gene was used as the endogenous reference control, and relative gene expression was determined using the 2^−ΔΔCT^ relative quantification method. To obtain a standard curve for the real-time PCR (RT-PCR), PCR was performed with each primer set by using calibrated amounts of chromosomal DNA; these reactions were performed at the same time as the qRT-PCR. The amplification efficiencies (E) of primer pairs used in the study were calculated from the standard curve obtained from a five-point 10-fold serial dilution series of cDNA template according to the following equation: E (%) = [10^(−1/slope)^ − 1] × 100. All experiments were performed with three biological replicates.

**TABLE 3:** Primers used in this study

Primer^*a*^	Sequence (5′–3′)^*b*^	Amplicon size (bp)	Amplication efficiency (%)^*c*^	Purpose
upF	TTAAGCTTGCTGGTCGTACTGCTGA	674		A1801 construct
upR	TCCATGCGAGCTCGAATTCGCATCTTCGACGTTTGCTTTG	674		
CmF	CAAAGCAAACGTCGAAGATGCGAATTCGAGCTCGCATGGA	851		
CmR	GGTTCTCCGGTGAGGACGCTTCGACGAATTTCTGCCATT	851		
downF	AATGGCAGAAATTCGTCGAAGCGTCCTCACCGGAGAACC	637		
downR	AAAGGATCCATCAGCCAGTCACCGAT	637		
testF	ATCTTGGGATTGCTGGGA	1,507		Validation of A1801 by PCR
testR	TTGTAGTTGGGATGCGGCT	1,507		Validation of A1801 by PCR
testSF	TGCTGAGAGTCGTTCCTA	900		Validation of A1901 by PCR
testSR	TGAAGCCACGAAAGGACA	900		Validation of A1901 by PCR
a15F	ATAGGATCCATTCATTGATTCGACTTC	641		pNfiR-wt construct
a15R	ATAAAGCTTGAGAGCAGGATGCGGTTG	641		pNfiR-wt construct
GSP1	CTTTGTCCGTGTCCCGCTCA			5′ RACE
GSP2	AACCTGCCGCTCCACGTTGA			5′ RACE
NorthernP	CTTTGTCCGTGTCCCGCTCAGTCGGGAAACCTGCCGCTCCACGTTGAGGGTCAG			[α-^32^P]-dCTP-labelled probes
RTnifDF	ACATGATCCACATTTCCCACG	197	99.7	qRT-PCR for the *nifD* half-life
RTnifDR	GAACAGCGTCTCGATCTCGTC	197	99.7	qRT-PCR for the *nifD* half-life
RTnifKF	TCGAGACCTACCTGGGCAACT	104	99.3	qRT-PCR for the *nifK* half-life
RTnifKR	GGGGTATCGAGCACTTCTTCC	104	99.3	qRT-PCR for the *nifK* half-life
RTnfiR-F	CTGACCCTCAACGTGG	54	99.9	qRT-PCR for the *nfiR* half-life
RTnfiR-R	CTTTGTCCGTGTCCCG	54	99.9	qRT-PCR for the *nfiR* half-life
FP-F	AGCGGTATTTCGAAGCCT	264		DNase I footprinting
FP-R	ATCTTCGACGTTTGCTTT	264		DNase I footprinting
RTnifAF	CGCGAAGACCTCTACTACCG	139	98.9	qRT-PCR
RTnifAR	CAGCTTGAGTTTGCGACCCT	139	98.9	qRT-PCR
RTnifHF	GAGATGATGGCGATGTATGC	113	99.1	
RTnifHR	GGTCGGTGTTGCGGCTGTTG	113	99.1	
RTnifDF	ACATGATCCACATTTCCCACG	197	99.7	
RTnifDR	GAACAGCGTCTCGATCTCGTC	197	99.7	
RTnifKF	TCGAGACCTACCTGGGCAACT	104	99.3	
RTnifKR	GGGGTATCGAGCACTTCTTCC	104	99.3	
RTrpoNF	CTTCTTCTCCAGCCACGTCAG	137	98.3	
RTrpoNR	CCAGTAAACCAGCGATCTTGC	137	98.3	
RTntrCF	GATCAATGGCGAATCGGGTAC	134	99.2	
RTntrCR	CAGCTCGGATTCCATCAGGTC	134	99.2	
RTglnKF	AGTCACTGCCATCATCAAGCC	183	99.7	
RTglnKR	GCCACGTCGATCTTCACCTTT	183	99.7	
RT16S-F	CCTACGGGAGGCAGCAG	160	99.8	
RT16S-R	ATTACCGCGGCTGCTGG	160	99.8	
RTnfiS-F	CCGCTGTCTGGCCTGTT	143	99.4	
RTnfiS-R	CCATGGGTGCCCGAATC	143	99.4	
RTncRNA05-F	CCAATACTCGGGGTTACGCT	175	98.3	
RTncRNA05-R	TGCCAAGCAGCAGGTCATAG	175	98.3	
RTncRNA08-F	CTCTTTCTGGGAGGTGGGTT	103	98.1	
RTncRNA08-R	CGGATACGGCAGTAGATAGTTTTA	103	98.1	
RTncRNA12-F	TCTTTCTGGGAGGTGGGTTA	102	98.6	
RTncRNA12-R	CGGATACGGCAGTAGATAGTTTTA	102	98.6	
RTcrcY-F	ATTGCCCGACAGGTTTCC	98	99.2	
RTcrcY-R	GACCATCGTCCGCATAGC	98	99.2	
RTncRNA30-F	TAAGCCTTTCGCCTCATCCA	92	98.8	
RTncRNA30-R	CATCATCCGTCTGTTGAAATCG	92	98.8	
RTncRNA31-F	CTGGTTTCAAAGATGTCGTGG	77	99.3	
RTncRNA31-R	ACGTCCCAGGCGGTCAGC	77	99.3	
RTncRNA32-F	GGGCACTACCAAGGCACG	141	98.5	
RTncRNA32-R	CGGTGAAGGCGGGTTTAG	141	98.5	
RTnfiR-F	CTGACCCTCAACGTGG	54	99.9	
RTnfiR-R	CTTTGTCCGTGTCCCG	54	99.9	
RTSRPbact-F	CGAGAAGGTCGTTATGGAGG	88	98.4	
RTSRPbact-F	GCGGGTTTCGTTATGGTG	88	98.4	
RTcrcZ-F	AGCAAAACAACGACAAGAAGG	184	99.7	
RTcrcZ-R	GGGAGCCAATAGCAAACG	184	99.7	
RTncRNA48-F	GGGGTTGCACTGCTCCAC	57	98.1	
RTncRNA48-R	CGCCTCATCCACCACAAG	57	98.1	
RTncRNA49-F	AAACTGCTTTTGGAGGTGCC	95	99.1	
RTncRNA49-R	GGCGAGGAGGAGTTGAGC	95	99.1	
RTncRNA50-F	CTGGTGGCGGAGACGAAG	91	98.3	
RTncRNA50-R	GTGGAATGGGGCTGGTTG	91	98.3	
RTncRNA51-F	TTGATGGTGTTCAGGGTTTTG	145	99.5	
RTncRNA51-R	GGTGCCGTTGTCGATGTTT	145	99.5	
RTncRNA53-F	ACAGGGATGTGGTGAATGC	122	98.5	
RTncRNA53-R	ACCCGCCCTACGGCTACT	122	98.5	
RTncRNA57-F	CAAGATGAACAGATGGACCGA	178	99.6	
RTncRNA57-R	GCACAGGAAACCAAGTAAAGC	178	99.6	

a F, forward; R, reverse.

b Restriction sites are underlined.

c The amplification efficiencies (E) of primer pairs used in the study were calculated from the standard curve obtained from a five-point 10-fold serial dilution series of cDNA template according to the following equation: E (%) = [10^(−1/slope)^ – 1] × 100.

### Nitrogenase activity assays.

Nitrogenase activity was determined with bacterial suspensions incubated to an OD_600_ of 0.1 in N-free minimal medium (0.5% oxygen and 10% acetylene) at 30°C (26). Protein concentrations were determined using a standard protein assay (Bio-Rad, Hercules, CA) with bovine serum albumin as a standard. The specific activity of nitrogenase was expressed as nmol ethylene per hour per milligram of protein. Each experiment was repeated at least three times.

### Abiotic stress-resistance assays.

Strain A1501 and mutant derivatives were grown in LB medium at 30°C to an OD_600_ of 0.6 and were then transferred into fresh LB medium in the presence or absence of 0.3 M sorbitol and 18 mM H_2_O_2_. At the time indicated (sorbitol stress, 1 h; H_2_O_2_ stress, 10 min), 10-fold serial dilutions were made, and 8 μl of each dilution was spotted onto solid LB plates. These plates were incubated at 30°C for 24 h before colony growth was observed and enumerated.

### Construction of the *nfiR* deletion mutant and complementation plasmids.

To generate the *nfiR* mutant strain A1801, a nonpolar deletion (covering residues from −10 to +54) was introduced into the Pseudomonas stutzeri chromosome by homologous recombination (19), as shown in Fig. S4. Amplification of a 674-bp DNA fragment located upstream of *nfiR* was performed using the primer set upF/upR, and amplification of a 637-bp DNA fragment located downstream of *nfiR* was performed using the set downF/downR (Table 3). Restriction enzyme sites (BamHI and HindIII) incorporated into the oligonucleotide primers to facilitate vector construction are underlined in the oligonucleotide sequences shown in Table 3. An 851-bp DNA fragment containing the Cm resistance cassette was amplified from the plasmid pKatCAT5 by PCR using the primers CmF and CmR. The three amplicons were fused into a 2.162-kb fragment in which the Cm gene is located between the other two amplicons by overlap extension PCR according to the strategy of PCR-based fusions (43). The fusion PCR product was then cloned into the multiple cloning site of the pMD18-T vector (TaKaRa, Japan). The resulting plasmid DNA was double digested with BamHI/HindIII and then cloned into the BamHI/HindIII site of pK18*mobsacB* (44). The resulting plasmid, pK18/delR, was mobilized from Escherichia coli into P. stutzeri strain A1501 by conjugation using pRK2013 (19) as the helper plasmid. After mating, cells were spread on LB plates containing 50 μg/ml Km and 40 μg/ml Cm to screen for clones in which pK18/delR had integrated into the A1501 genome via a single recombination event. Another recombination event was then induced to replace *nfiR* with *cat* and for removal of the Km^r^ and *sacB* genes from the genome. A colony of a single recombinant was then grown in a nonselective LB medium at 30°C. Cultures were diluted and spread onto LB agar supplemented with 10% (wt/vol) sucrose and 40 μg/ml Cm. The *nfiR* mutant strain was selected for kanamycin-sensitive and *sacB*-negative colonies. Correct recombination was checked using the primers testF and testR, followed by nucleotide sequencing of the amplicon obtained. The resulting *nfiR* deletion mutant, named A1801, was used for further study. The expression levels of flanking genes (*PST2408* and *PST2409*) were compared in the wild-type strain and the *nfiR* mutant by qRT-PCR. The experimental data indicated that *nfiR* deletion did not significantly alter the expression levels of the two flanking genes (data not shown). To explore the function of the NfiR stem-loop containing the 11-nt site that pairs with *nifD* mRNA, four 501-bp complementation DNA fragments carrying *nfiR* genes with different mutated sites, including sites within their promoter and terminator regions, were synthesized by BGI Co. Ltd. and cloned into the BamHI/HindIII site of pLAFR3 as described previously (19). The resulting four different complemented plasmids, named pNfiR-mut1, pNfiR-mut2, pNfiR-mut3, and pNfiR-mut4, were used for further studies.

### Construction of the double mutant strain lacking *nfiR* and *nfiS*.

A 2.2-kb fragment was double digested with BamHI/HindIII, in which the Spc gene is located between the upstream and downstream DNA fragments of *nfiS* by overlap extension PCR, and was then cloned into the BamHI/HindIII site of pK18*mobsacB*, generating pK18/delS (19). To engineer a Δ*nfiR* Δ*nfiS* double mutant, the homologous recombination plasmid pK18/delS was transformed into the genome of the *nfiR* mutant (A1801) by triparental mating, as described previously (19), and was chromosomally integrated upon first crossover selection for Km and Spc resistances. The second crossover cells were selected by culture on LB plates containing 10% (wt/vol) sucrose, spectinomycin (34 μg/ml), and chloramphenicol (40 μg/ml). Correct recombination was checked using the primers testSF and testSR (Table 3), followed by nucleotide sequencing of the amplicon obtained. The resulting Δ*nfiR* Δ*nfiS* double deletion mutant, named A1901, was used for further study.

### 5′ rapid amplification of cDNA ends.

The transcriptional start site of the *nfiR* gene was determined using 5′ rapid amplification of cDNA ends (5′ RACE) assay (Invitrogen, USA), following the manufacturer’s instructions. The sequences of the primers GSP1 and GSP2, which are specific for the *nfiR* gene tested here, are listed in Table 3. Products were cloned into a pMD18-T vector (TaKaRa, Japan) and sequenced to map the 5′ RACE end of the transcript.

### Measuring the stability of *nfiR*, *nifD*, and *nifK* transcripts.

To assay *nfiR, nifD*, and *nifK* mRNA stability, 2-ml bacterial samples incubated for 5 h under nitrogen fixation conditions were collected at different times (0, 1, 3, 5, and 7 min, or 0, 5, 10, 15, 20, 25, and 30 min) right after the addition of rifampin (400 μg/ml). Two volumes of RNAprotect (Sigma, USA) were added to each sample to prevent RNA degradation, and the samples were immediately frozen in liquid nitrogen. Total RNA was isolated from each sample for use in estimating mRNA levels by qRT-PCR. The primers used for measurement of the half-life of the selected gene mRNAs are listed in Table S2 in the supplemental material, and qRT-PCR was performed as described above. Data are presented as percent mRNA levels relative to time point zero.

### DNase I footprinting assay.

The DNA probe was prepared by PCR amplification of a 264-bp *nfiR* promoter region using the primers FP-F and FP-R (Table 3). The assay was performed in a total volume of 40 μl containing 500 ng of probe and various concentrations of purified NtrC protein. After incubation for 30 min at 25°C, 10 μl of solution, containing approximately 0.015 units of DNase I (Promega, USA) and 100 nmol of freshly prepared CaCl_2_, was added, and the sample was further incubated for 1 min at 25°C. The reaction was stopped by the addition of 140 μl of DNase I stop solution (200 mM unbuffered sodium acetate, 30 mM EDTA, and 0.15% sodium dodecyl sulfate [SDS]). Samples were first extracted with phenol-chloroform and then precipitated with ethanol, and the pellets were dissolved in 30 μl of Milli-Q water. The preparation of the DNA ladder, electrophoresis, and DNA sequencing were performed as described previously (45, 46).

### Microscale thermophoresis measurements.

MST experiments were performed according to Zhan et al. (19). A set of 30-nt ssRNA oligonucleotides containing wild-type or mutated base-pairing regions of NfiR (N-NfiR probes) or regions complementary to *nifD* mRNA (N-*nifD*R competitors) was synthesized by the GenePharma Company, and their sequences are listed in Table 4. The wild-type and mutated N-NfiR probe molecules were labeled with 6-carboxyfluorescein (FAM). Four microliters of sample, containing 500 nM labeled probe and increasing concentrations (from 10 nM to 300 μM) of a nonlabeled competitor, was loaded on standard treated silicon capillaries. Thermophoresis was monitored at 280 nm using a Monolith NT.115 instrument (NanoTemper Technologies GmbH), and the dissociation constants (*K_d_*) were calculated as previously described (19, 47). Data analyses were performed using NanoTemper Analysis software v.1.2.101 (NanoTemper Technologies, Germany).

**TABLE 4:** Synthesized ssRNA oligonucleotide derivatives for microscale thermophoresis

Name^*a*^	Sequence (5′–3′)^*b*^	Relevant characteristics
N-NfiR-wt	CAACUGACCCUCAA**CGUGGAGCGGC**AGGUU	WT, interaction with N-*nifD*-wt
N-NfiR-mut	CAACUGACCCUCAA**CCUCCAGCCCC**AGGUU	Mismatch mutation, no interaction with N-*nifD*R-wt
N-NfiR-com	CAACUGACCCUCAA**CGUAGAGCAGC**AGGUU	Compensatory mutation, stronger interaction with N-*nifD*R-wt
N-*nifD*R-wt	CAACGCCAC*GCUGCUCUACG*ACGACGUCAC	WT, interaction with N-NfiS-wt
N-*nifD*R-mut	CAACGCCAC*CGACGACUAGC*ACGACGUCAC	Mismatch mutation, no interaction with N-NfiR-wt
N-*nifD*R-com	CAACGCCAC*GCCGCUCCACG*ACGACGUCAC	Compensatory mutation, stronger interaction with N-NfiR-wt

a mut, mutation; N, 30-nt ssRNA oligonucleotide; com, compensatory mutation; *nifD*R, *nifD* mRNA; wt, wild type.

b The 11-nt sequence of the NfiR stem-loop pairing with *nifD* mRNA is shown in boldface. The 11-nt sequence of the complementary region at the 5′ end of *nifD* mRNA is shown in italics. Point mutations introduced into synthesized oligonucleotide derivatives are underlined.

### Northern blotting.

Total RNAs used for Northern blot analysis were prepared from P. stutzeri cells grown under nitrogen fixation conditions as described above using the SV total RNA isolation system (Promega, Madison, WI, USA). [α-^32^P]-dCTP-labeled probes were obtained as follows: PCR fragments of the *nfiR* gene were synthesized with P. stutzeri A1501 genomic DNA as the template and pairs of primers listed in Table 3. Ten micrograms of RNA was mixed with formaldehyde load dye, loaded into separate wells of a 1.5% Tris-acetate-EDTA (TAE)-agarose gel, and separated by electrophoresis. RNA was transferred to nylon membrane via capillary blotting, UV cross-linked, and probed overnight with an ncRNA-specific probe. Following hybridization and washing, Northern blots were developed and exposed to film.

### Western blot assays for NifDK expression.

Western blotting was performed using protein extracts of bacterial cells incubated for 5 h under nitrogen fixation conditions. A total protein loading control was used to quantify the expression levels of the target proteins in various samples. Protein concentrations were determined using the Bio-Rad protein assay reagent kit. Equal amounts of protein from each sample were separated by SDS polyacrylamide gel electrophoresis (SDS-PAGE) with an acrylamide/bisacrylamide ratio of 172:1 and were transferred to a polyvinylidene difluoride (PVDF) membrane (Amersham, USA) by electroblotting. The membrane was incubated with antisera raised against the MoFe protein (kindly provided by Ying Li, China Agricultural University, Beijing) for 2 h at 37°C and then washed three times in Tris-buffered saline (TBS)/Tween before being incubated with anti-rabbit secondary antibodies (Sangon, China) for 2 h. Detection was performed using an HRP-DAB chemistry kit (Tiangen, China).

### Proteomic analysis.

Bacterial cells were harvested and centrifuged at 8,000 × *g* for 5 min at 4°C. Protein samples were then purified by phenol saturated with Tris-HCl (pH 8.6) and precipitated with five volumes of 0.1 M ammonium acetate in methanol at −20°C overnight. Supernatant containing the soluble protein fraction was immediately subjected to two-dimensional polyacrylamide gel electrophoresis (2-DE) for protein separation, and each sample was analyzed in triplicate. For MALDI-TOF mass spectrometry (MS) analysis, protein spots were excised from gels and digested with trypsin as previously described (48), and the various peptides generated were analyzed using a 4700 proteomics analyzer (Applied Biosystems, USA). Proteins were identified by automated peptide mass fingerprinting using GPS Explorer v3.5 software (Applied Biosystems, USA). The identified differentially expressed proteins were functionally categorized using the Gene Ontology Tool. Gene ontology (GO) enrichment analysis was performed using GOEAST.

### Accession number(s).

The RNA-seq data described here have been deposited in the NCBI Sequence Read Archive (SRA) database under the accession numbers SRR8369128 and SRR8369127.

## Supplementary Material

Supplemental file 1
