# Supplementary material for: NfiR, a New Regulatory Noncoding RNA (ncRNA), Is Required in Concert with the NfiS ncRNA for Optimal Expression of Nitrogenase Genes in Pseudomonas stutzeri A1501
Source: Appl Environ Microbiol. 2019 Jul 1;85(14):e00762-19. doi: 10.1128/AEM.00762-19 (PMC6606865; doi:10.1128/AEM.00762-19)
Supplement: Supplemental file 1 [file AEM.00762-19-s0001.pdf]

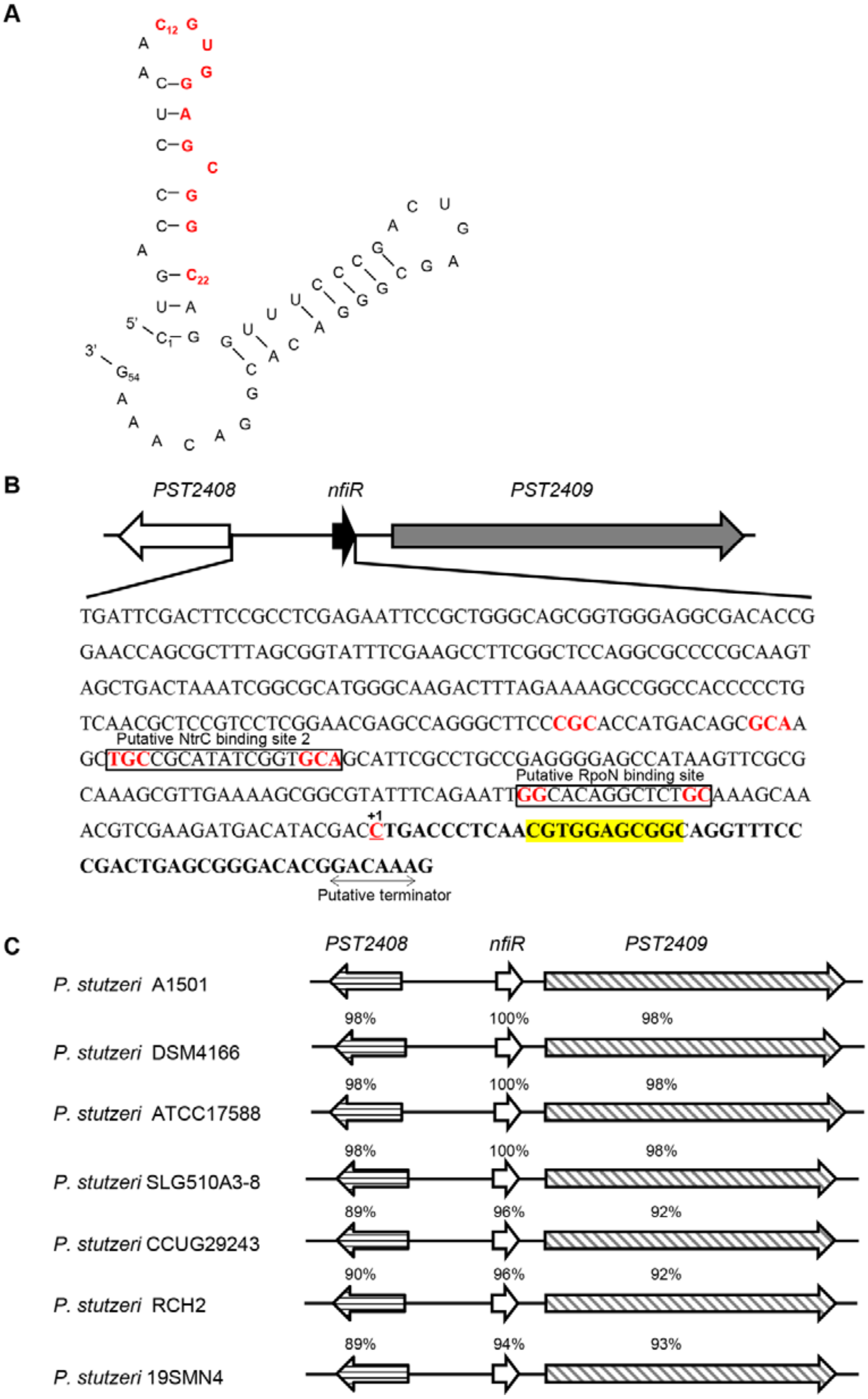

FIG S1 Identification and characterization of the *P. stutzeri* A1501 NfiR. (A) Secondary

structure of NfiR. The 11-nucleotide sequence predicted to be complementary to *nifD* mRNA is highlighted in red. (B) Physical map and nucleotide sequence of the *nfiR* region of *P. stutzeri* A1501. The nucleotide sequences, which may correspond to the predicted NtrC- or RpoN-binding sites, are boxed. +1, transcription start site mapped by 5' RACE; arrowheads, putative transcriptional terminator. The 11-nucleotide sequence predicted to be complementary to *nifD* mRNA is highlighted in yellow. (C) Genomic organization of the A1501 *nfiR* gene and a comparison with equivalent loci from other *Pseudomonas* species. The *nfiR* sequence is present in all sequenced *P. stutzeri* strains and is conserved at both the 5' and 3' ends of the genes. The numbers underneath the arrows indicate the percentage of amino acid sequence identity between A1501 NfiR and its homologs.

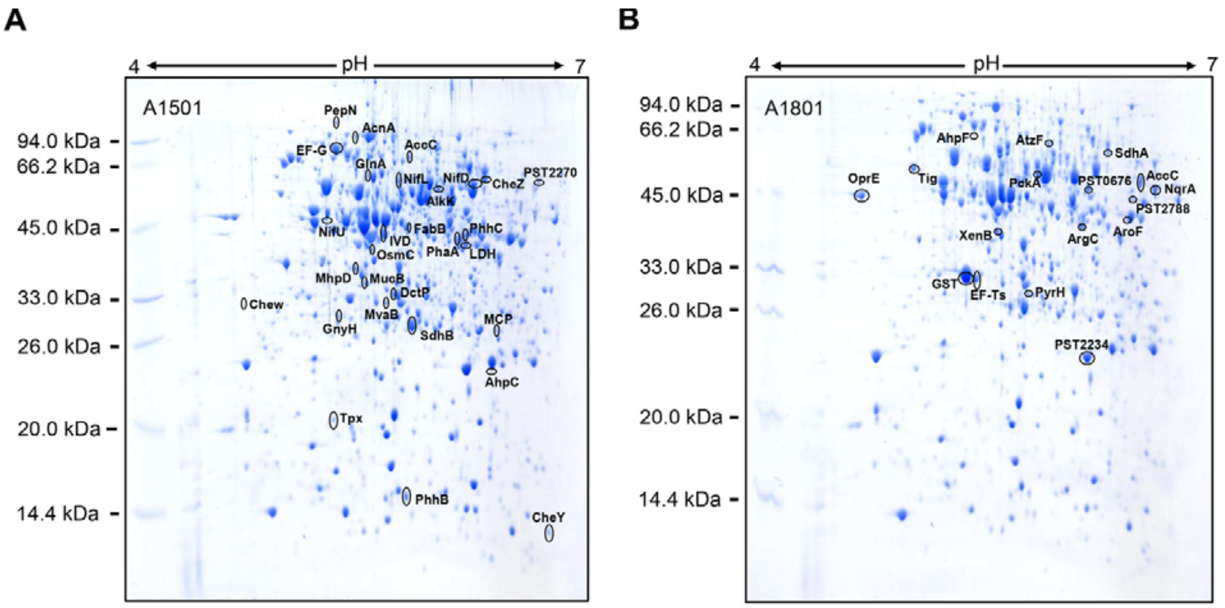

FIG S2 Protein profiles of wild-type A1501 (A) and the *nfiR* mutant (B) under nitrogen fixation conditions. Protein spots indicated by numbers are the differentially expressed proteins. All the labeled spots were identified by MALDI-TOF MS. Estimated molecular masses in kilodaltons are indicated by numbers at the left side of the gel image.

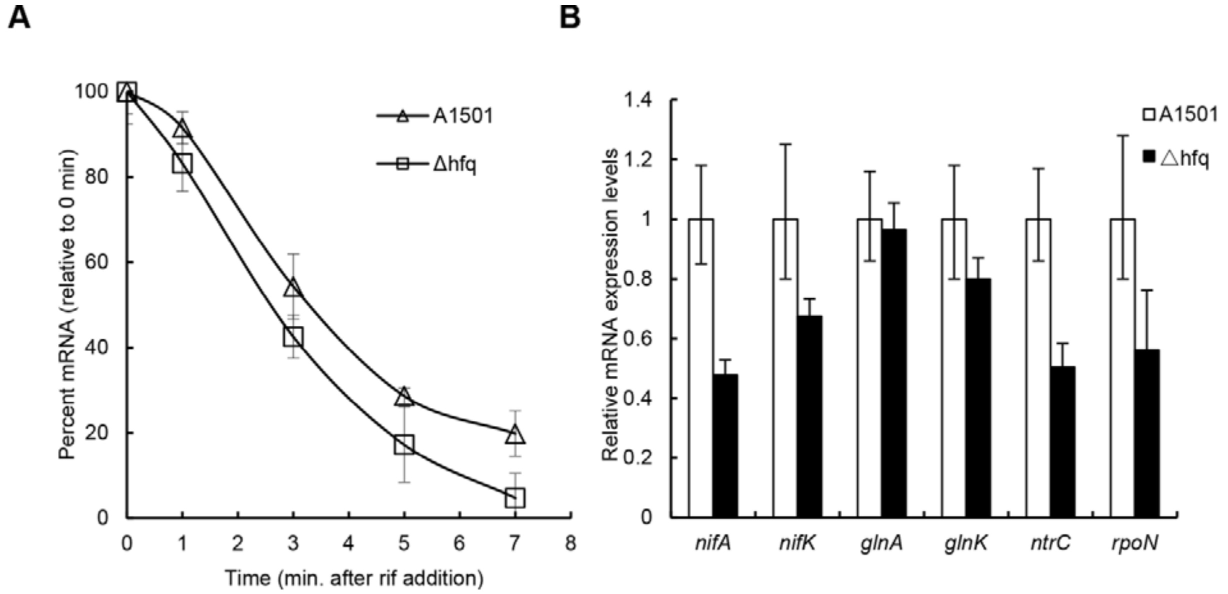

FIG S3 Effect of *hfq* mutation on the half-life of the *nfiR* transcript (A) and *nif* gene expression (B). Determination of the *nfiR* mRNA half-life in the wild-type A1501 and *hfq* mutant strains in the same conditions as Fig. 7. Data are the means and standard deviations of three independent experiments.

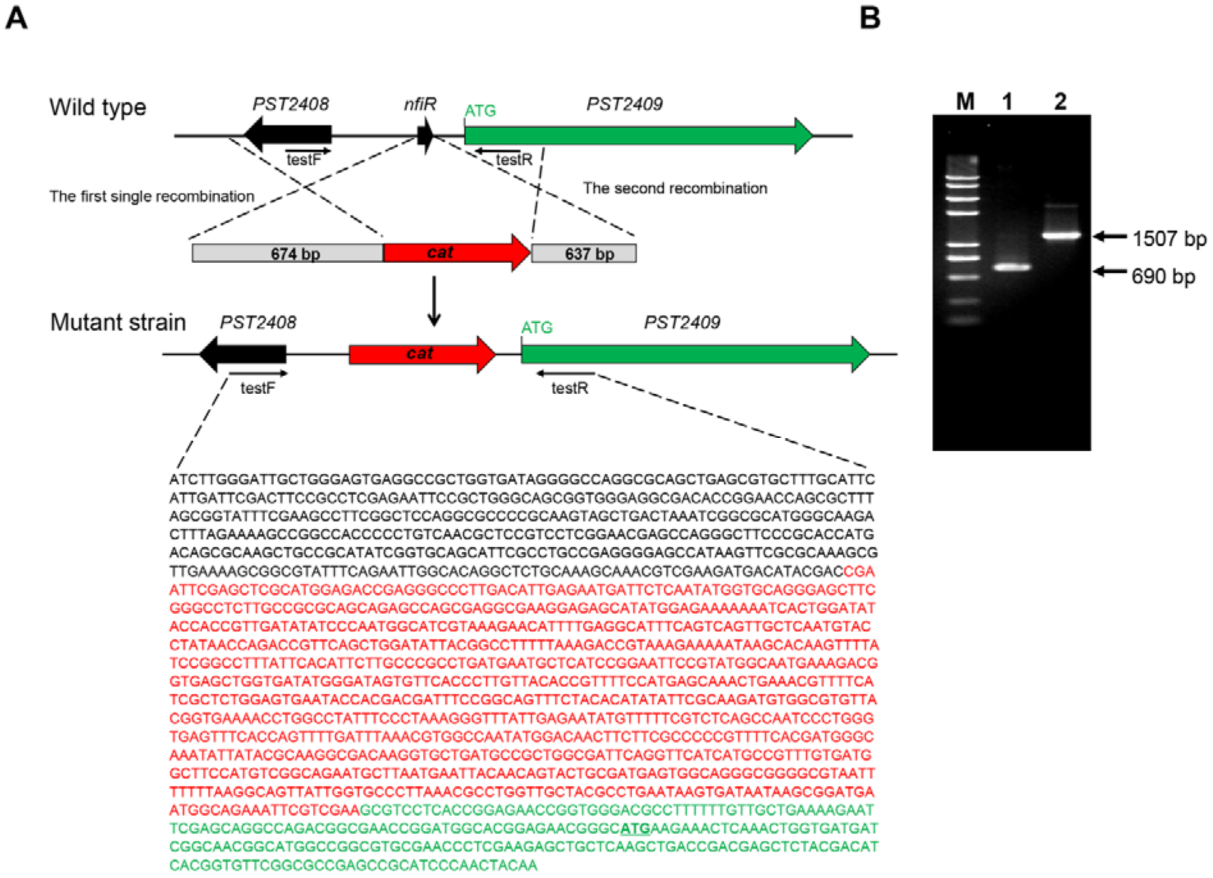

Fig. S4 Construction and verification of the Δ*nfiR* mutant. (A) Schematic representation of the Δ*nfiR* mutant generated by replacing the *nfiR* region with the chloramphenicol resistance gene *cat* (Cm^r). (B) PCR verification of the Δ*nfiR* mutant. Lane M: Trans2K PlusII DNA marker; lanes 1: PCR products amplified from the *P. stutzeri* A1501 WT using primers testF and testR; lanes 2: PCR products amplified from the Δ*nfiR* mutant using primers testF and testR.

TABLE S1 Small ncRNAs detected in *P. stutzeri* A1501 grown under nitrogen fixation conditions[a].

ID	NcRNA	Precicted length(nt)	Intergenic region	Rfam prediction	Conservation across other genomes[a]	Transcriptome fold change[b]
1	ncRNA01	254	PST30(+)-PST31(+)	NfiS	+	0.1
2	ncRNA05	186	PST0449(-)-PST0450(+)		+	0.3
3	ncRNA08	377	PST0570(+)-PST0571(+)		-	0.3
4	ncRNA12	372	PST0670(+)-PST0671(+)		-	0.4
5	ncRNA29	316	PST1920(+)-PST1921(+)	CrcY	++	0.1
6	ncRNA30	182	PST1943(-)-PST31(+)		+++	0.2
7	ncRNA31	119	PST1955(-)-PST1956(-)		+	0.01
8	ncRNA32	219	PST2072(+)-PST2073(+)		++	0.3
9	ncRNA34	54	PST2408(-)-PST2409(+)	NfiR	+	0.04
10	ncRNA35	191	PST2513(-)-PST2514(-)	SRP bact	+++	0.4
11	ncRNA45	67	PST3289(+)-PST3290(+)		+	0.3
12	ncRNA46	340	PST3293(+)-PST3294(+)	CrcZ	++	0.2
13	ncRNA49	712	PST3751(+)-PST3752(-)		+++	0.4
14	ncRNA50	522	PST3751(+)-PST3752(-)		+++	0.3
15	ncRNA51	381	PST3751(+)-PST3752(-)		+++	0.3
16	ncRNA53	246	PST3824(+)-PST3825(-)		-	0.3
17	ncRNA57	356	PST4127(+)-PST4128(-)		+	0.3
18	ncRNA19	342	PST1169(-)-PST1170(-)		+	3.3
19	ncRNA21	168	PST1236(-)-PST1237(-)		+	2.5
20	ncRNA39	25	PST2989(+)-PST2990(-)		-	2.1
21	ncRNA42	224	PST3227(+)-PST3228(-)		+	5.0
22	ncRNA48	70	PST3745(+)-PST3746(+)		-	10
23	ncRNA55	239	PST4033(-)-PST4034(-)		+	2.5
24	ncRNA02	38	PST0067(+)-PST0068(+)		+	0.5

25	ncRNA03	115	PST0113(+)-PST0115(-)		++	0.6
26	ncRNA04	324	PST0217(-)-PST0218(-)		-	1.5
27	ncRNA06	39	PST0523(+)-PST0524(+)		+	1.2
28	ncRNA09	1150	PST0635(-)-PST0636(+)		-	0.7
29	ncRNA10	570	PST0639(+)-PST0640(+)		-	1.8
30	ncRNA13	543	PST0848(-)-PST0849(-)		+	0.6
31	ncRNA15	86	PST0991(+)-PST0992(-)		+	0.9
32	ncRNA16	115	PST0991(+)-PST0992(-)		+	1.6
33	ncRNA17	366	PST1072(+)-PST1073(+)	Rnase P	+++	1.1
34	ncRNA18	189	PST1115(-)-PST1116(-)		+++	1.1
35	ncRNA20	481	PST1212(-)-PST1213(-)		+	1.0
36	ncRNA22	181	PST1276(+)-PST1277(+)	P15	++	0.8
37	ncRNA24	173	PST1412(-)-PST1413(-)		-	1.1
38	ncRNA25	428	PST1498(-)-PST1499(+)		+	1.6
39	ncRNA26	167	PST1572(+)-PST1573(+)	RSmZ	++	1.6
40	ncRNA27	639	PST1763(-)-PST1764(-)		+	0.8
41	ncRNA28	487	PST1818(+)-PST1819(+)		+	1.9
42	ncRNA33	80	PST2124(+)-PST2125(+)		+	1.8
43	ncRNA36	303	PST2630(-)-PST2631(+)		+++	1.3
44	ncRNA37	222	PST2714(+)-PST2715(+)		+	0.5
45	ncRNA38	240	PST2880(-)-PST2881(-)		+	0.7
46	ncRNA40	224	PST3173(-)-PST3174(-)		-	1.0
47	ncRNA41	394	PST3203(-)-PST3204(-)		+	1.4
48	ncRNA43	168	PST3229(-)-PST3230(-)		+	1.2
49	ncRNA44	20	PST3248(-)-PST3249(-)		-	0.6
50	ncRNA47	116	PST3715(+)-PST3716(+)		+	1.6
51	ncRNA52	167	PST3816(-)-PST3817(+)		+	1.0

52	ncRNA54	231	PST3987(+)-PST3988(+)		-	0.6
53	ncRNA56	179	PST4072(+)-PST4073(+)	6SRNA	+++	0.6

[a]The BLASTN searches of the 53 ncRNAs that were predicted in *P. stutzeri* A1501 under nitrogen fixation conditions against the GenBank database by using Solexa/Illumina sequencing at Tianjin Biochip Corporation (Tianjin, China) showed that 12 were specific to A1501 (-); 26 ncRNAs were found in only some *Pseudomonas stutzeri* strains (+); and 15 were conserved in other species of *Pseudomonas* (++) or in other bacteria (+++). Only eight ncRNAs (NfiS, CrcZ, CrcY, RnaseP, P15, Rsmz, SRP bact and 6SRNA) have been previously characterized, and the functions of the rest of the ncRNAs are still unknown. This catalog of candidate regulatory ncRNAs will serve as an important reference point for comprehensive analyses of ncRNA regulation in *P. stutzeri* and other *Pseudomonas* species.

[b]Induction ratio (ammonium shock conditions/nitrogen fixation conditions) after exposure of bacteria to 20 mM ammonia for 10 min.

TABLE S2 Identification of proteins from the selected spots by MALDI-TOF-MS.

Locus tag	Gene	Gene product	PI/Mass(kDa)(theoretical)	Fold change in Δ*nfiR* vs. wild type[a]
**Amino acid metabolism-related protein**				
PST2762	*atzF*	allophanate hydrolase	5.91/61.3	5.96
PST2024	*aroF-1*	phospho-2-dehydro-3-deoxyheptonate aldolase	6.55/40.9	3.47
PST0676	_	arginine deiminase	6.22/46.8	2.66
PST3274	*accC*	acetyl-CoA carboxylase biotin carboxylase subunit	6.66/49.1	2.22
PST0753	*argC*	N-acetyl-gamma-glutamyl-phosphate reductase	6.17/39.8	2.02
PST0934	*gdhA*	glutamate dehydrogenase	6.43/45.6	1.09
PST3215	*gnyH*	γ-carboxygeranoyl-CoA hydratase	5.24/28.8	NA[b]
PST3165	*argJ*	bifunctional ornithine acetyltransferase/N-acetylglutamate synthase	5.18/42.9	NA[b]
PST1222	*nadB*	L-aspartate oxidase	5.69/64.7	NA[b]
PST2005	_	allophanate hydrolase	5.54/64.5	NA[b]
PST2819	*proS*	prolyl-tRNA synthetase	5.36/63.6	NA[b]
PST0193	*ansA*	L-asparaginase I	6.14/39.8	NA[b]
PST1277	*aroF-2*	phospho-2-dehydro-3-deoxyheptonate aldolase	6.06/39.3	NA[b]
PST1772	*leuC*	isopropylmalate isomerase large subunit	5.54/51.6	0.83
PST2895	*trpA*	tryptophan synthase subunit beta	6.01/44.4	0.58
PST3213	*ivD*	isovaleryl-CoA dehydrogenase	5.59/41.9	0.47
PST2998	*aspC*	aromatic amino acid aminotransferase	6.2/41.8	0.41
PST0867	*ldh*	leucine dehydrogenase	6.21/39.8	0.39
PST2788	_	phosphoribosylaminoimidazole-succinocarboxamide synthase	5.24/32.2	0.35
PST3608	*add*	adenosine deaminase	4.91/35.7	0.34
PST4064	*gcvT*	aminomethyltransferase	4.84/28.1	0.23
PST3564	*phhC*	aromatic aminoacid aminotransferase	5.77/43.8	0.22
PST1774	*leuB*	3-isopropylmalate dehydrogenase	4.68/38.7	0.21
PST0871	_	2-keto-4-pentenoate hydratase	5.13/35.8	0.14

PST0568	*metY*	homocysteine synthase	5.81/45.8	0.19
PST1716	*mvaB*	hydroxymethylglutaryl-CoA lyase	5.61/31.2	0.11
PST2349	*dcyD*	D-cysteine desulfhydrase	6.67/39.1	0.08
PST0869	*hpd*	4-hydroxyphenylpyruvate dioxygenase	5.13/40.6	0.05
PST3178	_	NAD-dependent aldehyde dehydrogenase	5.17/17.1	NA[C]
PST1258	*pcaD*	beta-ketoadipate enol-lactone hydrolase	5.23/28.5	NA[C]
PST1877	*lpdG*	dihydrolipoamide dehydrogenase	5.69/50.1	NA[C]
PST0060	*arcA*	arginine deiminase	5.77/46.4	NA[C]
PST0684	*phaA*	acetyl-CoA acetyltransferase	6.19/40.7	NA[C]
PST3003	*argF*	ornithine carbamoyltransferase	6.58/42.6	NA[C]
PST1225	*mucB*	negative regulator for alginate biosynthesis	5.46/34.5	NA[C]
PST1743	_	tryptophan synthase subunit beta chain	6.01/44.3	NA[C]
**Central carbon and nitrogen metabolism-related proteins**				
PST1873	*sdhA*	succinate dehydrogenase flavoprotein subunit	6.37/56.8	2.84
PST0897	*narH*	respiratory nitrate reductase beta chain	5.9/59.0	1.41
PST1377	_	oxaloacetate decarboxylase	5.02/64.1	NA[b]
PST2797	_	acetyl-CoA hydrolase	5.66/53.7	NA[b]
PST2265	_	quinoprotein alcohol dehydrogenase	9.02/71.8	NA[b]
PST3532	*nirS*	cytochrome cd1 nitrite reductase	6.72/61.9	NA[b]
PST1350	nifU	Fe-S cluster assembly protein	4.96/34.3	0.85
PST3898	_	malate synthase G	5.13/79.6	0.69
PST2671	*cysM*	cysteine synthase B	5.77/35.2	0.53
PST2036	*acnA*	aconitate hydratase	5.37/100	0.48
PST0353	*glnA*	glutamine synthetase	5.48/58.6	0.46
PST1314	*nifL*	nitrogen fixation negative regulatory protein	5.72/57.1	0.45
PST3220	_	acetyl-CoA acetyltransferase	6.15/40.9	0.43
PST0550	*maeB*	malic enzyme	5.12/45.3	0.4
PST1327	*nifD*	MoFe protein alpha subunit	6.26/56.8	0.39
PST2270	_	quinoprotein alcohol dehydrogenase,	6.81/58.1	0.34
PST0494	*mmsA*	methylmalonate-semialdehyde dehydrogenase	5.21/53.9	0.31
PST1846	*acnA*	aconitate hydratase 1	5.14/97.4	0.28

PST3257	*ilvH*	acetolactate synthase 3 regulatory subuni	5.93/17.8	0.26
PST1874	*sdhB*	succinate dehydrogenase iron-sulfur subunit	5.83/26.3	0.2
PST1268	*napA*	nitrate reductase catalytic subunit	6.43/94.1	0.2
PST4048	_	TRAP-type transport system, periplasmic protein	5.93/34.9	0.18
PST0293	*pckA*	phosphoenolpyruvate carboxykinase	5.84/50	NA[b]
PST1621	*gnl*	gluconolactonase	5.67/43.5	NA[b]
PST1328	*nifK*	MoFe protein beta subunit	5.62/60.1	NA[C]
PST1512	_	phosphoketolase	5.90/89.1	NA[C]
PST2035	*prpC*	methylcitrate synthase	6.03/42.1	NA[C]
**Chemotaxis and cell mobility-related proteins**				
PST2567	*cheZ*	chemotaxis protein	4.61/29.9	0.31
PST2560	*cheW*	chemotaxis protein	4.3/17.7	0.11
PST2199	_	putative ABC transporter periplasmic solute-binding protein	5.11/37.3	NA[C]
PST2110	*mCP*	methyl-accepting chemotaxis protein	7.81/32.3	NA[C]
PST2568	*cheY*	two-component response regulator	6.87/13.4	NA[C]
**Toxic, stress response or other metabolism-related proteins**				
PST3481	*gST*	glutathione S-transferase	5.24/33.1	60.6
PST2142	*glgX*	glycogen operon protein	5.32/81.9	20.9
PST3339	_	hypothetical protein	5.35/24.7	15.7
PST1565	*xenB*	xenobiotic reductase	5.5/38.6	13.6
PST2060	*tig*	trigger factor	4.83/54.7	4.13
PST2818	*oprE3*	outer membrane protein, OprE3	4.49/45.6	3.51
PST2234	_	HSP20 family protein	6.21/23.5	2.88
PST0817	*oprG*	outer membrane protein	4.73/23.9	2.67
PST2214	_	hypothetical protein	6.7/12.2	2.66
PST2655	*nqrA*	Na(+)-translocating NADH-quinone reductase subunit A	6.77/47	2.61
PST3323	*carB*	carbamoyl phosphate synthase large subunit	5.05/118.5	1.65
PST1336	_	hypothetical protein	5.4/17.7	1.32
PST1539	*pyrH*	uridylate kinase	5.74/29.2	NA[b]
PST4067	*ubiF*	benzoquinol hydroxylase	6.59/44.4	NA[b]
PST2334	_	glutathione S-transferase family protein	5.09/31.2	NA[b]
PST3431	_	MerR family transcriptional regulator	5.38/34.8	NA[b]
PST1602	*glpR*	glycerol-3-phosphate regulon repressor	5.5/27.9	NA[b]

PST3070	*ndpA*	nucleoid-associated protein	5.9/42.4	NA[b]
PST1968	*hemH*	ferrochelatase	5.96/38.8	NA[b]
PST3161	*ahpF*	alkyl hydroperoxide reductase subunit F	5.36/56.3	NA[b]
PST0389	*phoP*	two-component response regulator	5.16/27.3	NA[b]
PST1051	*cysN*	bifunctional sulfate adenylyltransferase subunit	5.55/69.4	0.92
PST1227	*mucD*	serine protease	5.65/49.8	0.92
PST1088	*lpxC*	UDP-3-O-acyl-N-acetylglucosamine deacetylase	4.82/30.0	0.88
PST4053	*algB*	alginate biosynthesis transcriptional regulator	5.61/49.6	0.84
PST1892	*fabB*	3-oxoacyl-ACP synthase	5.81/42.9	0.46
PST2415	*pepN*	aminopeptidase N	5.17/109	0.45
PST1918	*alkK-2*	medium-chain-fatty-acid--CoA ligase	6.02/53.9	0.41
PST0781	*eF-G*	elongation factor G	5.21/84	0.41
PST0078	*dctP*	C4 dicarboxylate binding protein	5.68/32	0.41
PST3563	*phhB*	pterin-4-alpha-carbinolamine dehydratase	5.79/15.1	0.38
PST4121	_	short-chain dehydrogenase	5.78/32.7	0.33
PST1498	_	hypothetical protein	5.09/36.5	0.27
PST2181	*tpX*	thiol peroxidase	5.2/20.6	0.26
PST2354	_	luciferase family protein	5.02/39.5	0.23
PST2276	_	TonB-dependent receptor	5.20/84.3	0.22
PST1912	_	hypothetical protein	5.83/18.1	0.22
PST2272	_	pentapeptide repeat-containing protein	5.31/23.5	0.11
PST3941	_	YceI-like family protein	5.3/20.4	0.04
PST2868	_	glyoxalase/bleomycin resistance protein/dioxygenase	6.58/17.4	0.03
PST2440	_	binding protein component of ABC sugar transporter	5.67/48.5	0.02
PST3173	*aidA*	type V secretory pathway, adhesion	4.91/65.2	0.01
PST2271	*exaB*	cytochrome c550	4.88/15.9	NA[C]
PST1850	_	cytochrome C oxidase	4.87/20.5	NA[C]
PST2608	_	hypothetical protein	4.82/20.8	NA[C]
PST2269	_	periplasmic binding protein	5.76/33.5	NA[C]
PST1538	*eF-Ts*	elongation factor Ts	5.16/30.6	NA[C]
PST2824	*arsC-1*	arsenate reductase	6.58/16.6	NA[C]
PST0265	*osmC*	osmotically inducible protein	5.67/15.1	NA[C]
PST0369	_	chaperone ATPase	5.97/17.6	NA[C]

PST3148	_	putative heme iron utilization protein	6.11/26.2	NA[C]
PST3162	*ahpC*	alkyl hydroperoxide reductase subunit C	5.9/20.8	NA[C]
PST3222	_	transporter, periplasmic protein	6.17/40.3	NA[C]
PST3235	_	alpha, alpha-trehalose-phosphate synthase	5.95/54.0	NA[C]

[a]Mean of three independent measurements.

[b]Ratio could not be measured because the protein spot was undetectable in the wild-type strain.

[c]Ratio could not be measured because the protein spot was undetectable in the Δ*nfiR* mutant.
